# *Campylobacter jejuni* resistance to human milk involves the acyl carrier protein AcpP

**DOI:** 10.1128/mbio.03997-24

**Published:** 2025-02-25

**Authors:** Bibi Zhou, Jolene M. Garber, James Butcher, Artur Muszynski, Rebekah L. Casey, Steven Huynh, Stephanie Archer-hartmann, Sara Porfírio, Ashley M. Rogers, Parastoo Azadi, Craig T. Parker, Kenneth K. S. Ng, Kelly M. Hines, Alain Stintzi, Christine M. Szymanski

**Affiliations:** 1 Department of Microbiology, University of Georgia, Athens, Georgia, USA; 2 Complex Carbohydrate Research Center, University of Georgia, Athens, Georgia, USA; 3 School of Pharmaceutical Sciences, Ottawa Institute of Systems Biology and Department of Biochemistry, Microbiology and Immunology, Faculty of Medicine, University of Ottawa, Ottawa, Ontario, Canada; 4 Department of Chemistry, University of Georgia, Athens, Georgia, USA; 5 Produce Safety and Microbiology Research Unit, Agricultural Research Service, U.S. Department of Agriculture, Albany, California, USA; 6 Department of Chemistry and Biochemistry, University of Windsor 8637 , Windsor, Ontario, Canada; Fred Hutchinson Cancer Center, Seattle, Washington, USA

**Keywords:** *Campylobacter jejuni*, human milk, AcpP, PorA, EptA, antimicrobial peptides

## Abstract

*Campylobacter jejuni* is a common foodborne pathogen worldwide that is associated with high rates of morbidity and mortality among infants in low- to middle-income countries (LMICs). Human milk provides infants with an important source of nutrients and contains antimicrobial components for protection against infection. However, recent studies, including our own, have found significantly higher levels of *Campylobacter* in diarrheal stool samples collected from breastfed infants compared to non-breastfed infants in LMICs. We hypothesized that *C. jejuni* has unique strategies to resist the antimicrobial properties of human milk. Transcriptional profiling found human milk exposure induces genes associated with ribosomal function, iron acquisition, and amino acid utilization in *C. jejuni* strains 81–176 and 11168. However, unidentified proteinaceous components of human milk prevent bacterial growth. Evolving both *C. jejuni* isolates to survive in human milk resulted in mutations in genes encoding the acyl carrier protein (AcpP) and the major outer membrane porin (PorA). Introduction of the PorA/AcpP amino acid changes into the parental backgrounds followed by electron microscopy showed distinct membrane architectures, and the AcpP changes not only significantly improved growth in human milk, but also yielded cells surrounded with outer membrane vesicles. Analyses of the phospholipid and lipooligosaccharide (LOS) compositions suggest an imbalance in acyl chain distributions. For strain 11168, these changes protect both evolved and 11168∆*acpP*^G33R^ strains from bacteriophage infection and polymyxin killing. Taken together, this study provides insights into how *C. jejuni* may evolve to resist the bactericidal activity of human milk and flourish in the hostile environment of the gastrointestinal tract.

**IMPORTANCE:**

In this study, we evolved *C. jejuni* strains which can grow in the presence of human milk and found that cell membrane alterations may be involved in resistance to the antimicrobial properties of human milk. These bacterial membrane changes are predominantly linked to amino acid substitutions within the acyl carrier protein, AcpP, although other bacterial components, including PorA, are likely involved. This study provides some insights into possible strategies for *C. jejuni* survival and propagation in the gastrointestinal tract of breastfed infants.

## INTRODUCTION

*Campylobacter jejuni* is the most common cause of bacterial foodborne illness in the USA (1). Worldwide, *C. jejuni* is among the top enteric pathogens isolated from patients with bacterial-induced diarrhea, with infections leading to high rates of morbidity and mortality in low- to middle-income countries (LMICs). This is particularly of concern in infants, where up to 85% of children from LMICs are *C. jejuni* stool positive by the age of 1 (2). Alarmingly, post-infectious complications such as environmental enteric dysfunction (EED), Guillain-Barré syndrome (GBS), reactive arthritis, and irritable bowel syndrome continue to increase. EED is a subclinical chronic disorder resulting from improper nutrient absorption, intestinal injury, and prolonged inflammation that leads to growth stunting, decreased oral vaccine responses, impaired cognitive development, and higher risk for the development of metabolic syndrome and its related cardiovascular sequelae (2).

Exclusive breastfeeding, clean drinking water, improved latrine, and appropriate antibiotic treatment have been reported to be important for reducing the risk of *Campylobacter* infection (3). Human milk has many benefits, including providing nutrition for the infants, assisting in the development of the intestinal microbiome, immune system, brain, and digestive system, and supplying antimicrobial components capable of protecting against infectious diseases (4–6). These antimicrobial components include lysozyme, which hydrolyzes the repeating sugar units within exposed peptidoglycan chains in gram-positive microbes; lactoferrin, a multifunctional protein which is important for sequestering iron from pathogens and binding to bacterial lipopolysaccharides (LPSs) in gram-negative microbes; and antibodies, oligosaccharides, fats, and antimicrobial peptides (AMPs), such as the cathelicidin LL-37 (7–12). The majority of AMPs in human milk stem from highly abundant protease-digested proteins including casein, lactalbumin, lactoferrin, and LL-37, the latter derived from its precursor (CAP-18), which needs to be cleaved by host proteases to release the cationic antimicrobial peptide; although several AMPs in freshly expressed whole milk do not require intestinal digestion for their release (6, 10). Several lipids from human milk can also possess antimicrobial activities that are activated when lipolytic enzymes derived from the host or milk commensal microbes convert fats such as triglycerides to antimicrobial fatty acids (FAs) and monoglycerides. Examples of active lipids include eicosapentaenoic acid, docosahexaenoic acid, and glycerol monolaurate (13, 14).

A recent focus of study has been examining the role of human milk oligosaccharides (HMOs) in shaping *C. jejuni* colonization and infection. HMOs and antibodies can block bacterial attachment to host surfaces, and antibodies can also signal phagocytosis by immune cells. HMOs are a group of glycans with different structures that are also highly abundant in human milk (15). In colostrum, the concentration of HMOs reaches 20–25 g/L, and as the mother’s milk matures, the concentration drops to 5–20 g/L (15, 16). HMOs can be extensively fucosylated, depending on the mother’s secretor status, and fucosylated HMOs have been proposed to act as decoys for pathogens and protect against infection (17, 18). Yu and colleagues reported that 2′-fucosyllactose can inhibit *C. jejuni* invasion *in vitro* and *in vivo* (17). Furthermore, many gut commensals can digest HMOs, including microbes such as *Bifidobacterium* and *Bacteroides*, and secrete fucosidases releasing up to 4–5 mg L-fucose from fucosylated HMOs per gram feces from breastfed infants (19–21). More than 60% of *C. jejuni* isolates can metabolize L-fucose and possess the ability to chemotax toward L-fucose (22). In addition, we have demonstrated that L-fucose metabolism enhances *C. jejuni* intestinal colonization in neonatal colostrum-deprived piglets (22, 23).

However, through genomics studies examining *Campylobacter* infection profiles in infants from seven sites in sub-Saharan African and South Asia, we discovered that exclusively breastfed infants showed a significantly higher abundance of *C. jejuni* in their stools compared to non-breastfed infants (24), and this has been confirmed by other studies (25, 26). Even more compelling was the observation that breastfed infants were predominantly colonized with non-L-fucose metabolizing *Campylobacter* strains, while infection with L-fucose-metabolizing *Campylobacters* was less frequent (24).

This led us to discover an energy taxis system that we hypothesized directs L-fucose-metabolizing *C. jejuni* to remain in the intestinal lumen exposed to unfavorably low levels of oxygen (27) and the propulsive action of gut peristalsis. In contrast, non-L-fucose metabolizing *C. jejuni* isolates lack the ability to sense L-fucose and would colonize the intestinal crypts of breastfed infants and catabolize the amino acids and metabolic by-products released by commensal microbes present during this time period of life.

Even though asaccharolytic *C. jejuni* isolates colonize the mucus layer and deep within the intestinal crypts away from human milk antimicrobial components, such as AMPs that are present in high concentrations in the lumen, low concentrations of AMPs can still diffuse through the mucosa. We hypothesized that *C. jejuni* may have some unique strategies to survive human milk in the gut of breastfed infants. In this study, we evolved two *C. jejuni* strains to grow in high concentrations of human milk. Both strains showed amino acid changes in the acyl carrier protein (AcpP), and the major outer membrane protein (PorA). Further studies indicated that the mutations in AcpP play the dominant role in *C. jejuni* human milk survival. We next used electron microscopy to demonstrate that the cell membrane of the evolved strains showed significant differences in morphology compared with the parental strains. Furthermore, the evolved strains were more resistant to polymyxin B, an antibiotic that disrupts bacterial outer membranes. Finally, we determined that proteinaceous factors within human milk are responsible for *C. jejuni* growth inhibition.

## RESULTS

### Incubation of *C. jejuni* with human milk leads to increases in cell metabolism, but the microbes cannot persist in this environment

**Fig 1:**
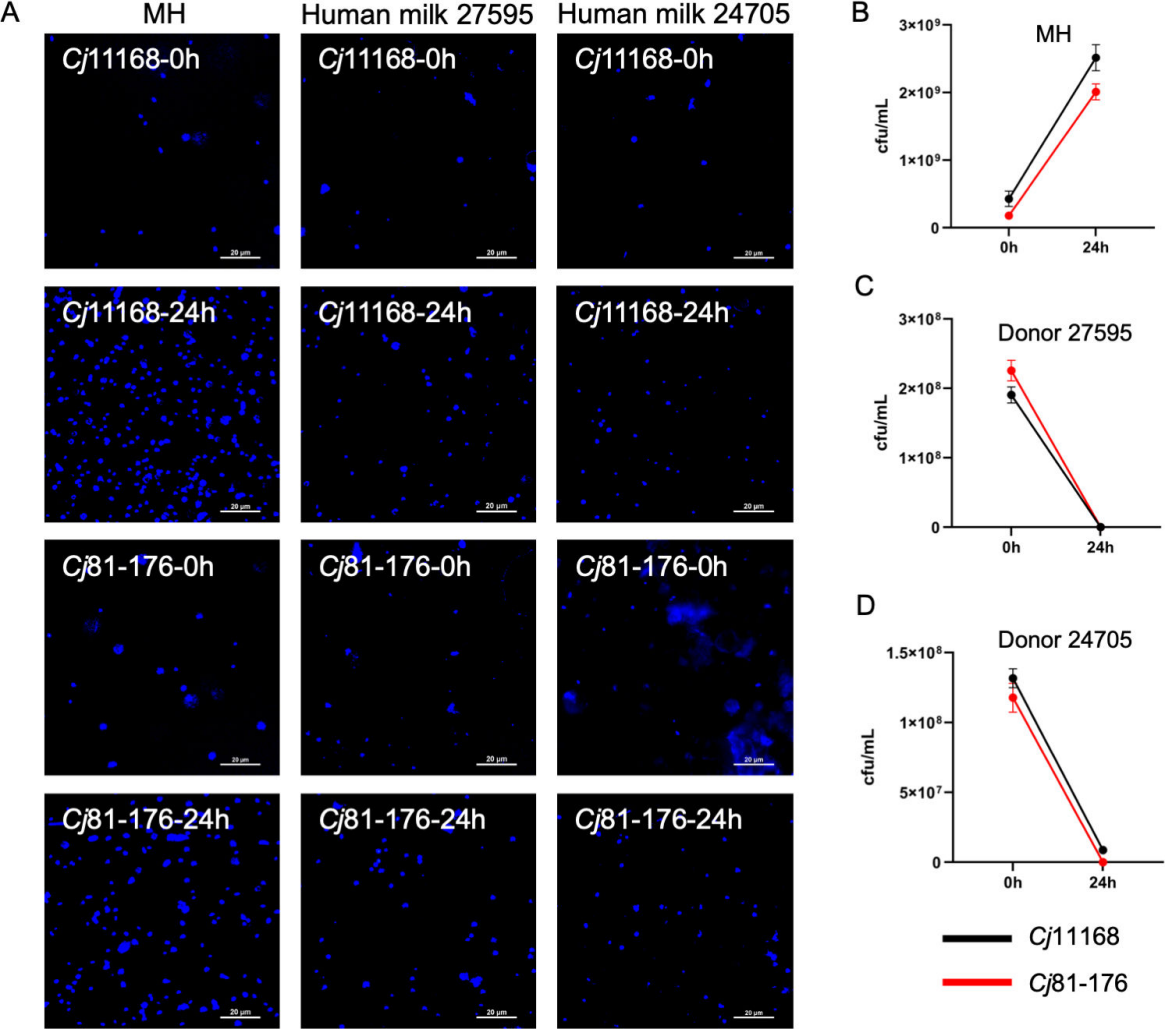
Growth of *C. jejuni* 11168 and *C. jejuni* 81–176 in MH or 100% human milk (donors 27575 and 24705). (**A**) Bacteria were stained with 4′,6-diamidino-2-phenylindole and observed by fluorescence microscopy at times 0 and 24 h. (**B–D**) Growth curves of *C. jejuni* 11168 and *C. jejuni* 81–176 in MH (**B**), and 100% human milk from donor 27575 (**C**) and donor 24705 (**D**) at times 0 and 24 h. Microscopy images are representative of three experiments, and error bars in panels B–D represent the standard error of the mean from three separate biological experiments.

Given that there are increasing reports describing the prevalence of *C. jejuni* infection in breastfed infants, we attempted to grow *C. jejuni* 11168 and 81–176 in 100% human milk (donors 27595 and 24705). Strain 11168 was used as a representative fucose-metabolizing strain, while 81–176 was used as a non-fucose-metabolizing strain, since we initially hypothesized that the presence of fucosylated HMOs may lead to different growth phenotypes. After 24 h incubation, 4′,6-diamidino-2-phenylindole-stained cells were observed using fluorescence microscopy (Fig. 1A). Both strains showed increased growth in Mueller-Hinton (MH) broth that was confirmed by comparing colony counts at 0 and 24 h (Fig. 1A and B). After 24 h incubation in 100% human milk, there appeared to be only slightly more cells compared to the 0 h time point (Fig. 1A), however, colony counts after 24 h indicated that both strains showed a dramatic drop in numbers (Fig. 1C and D). These results suggested that both *C. jejuni* strains were capable of a limited number of cell divisions in 100% human milk, but were ultimately killed by human milk.

To investigate how human milk affects *C. jejuni* gene expression, we incubated *C. jejuni* 11168 and 81–176 with human milk (donors 27595 and 24705) for 15 min and analyzed the resulting transcriptomes (from three to four replicates) compared to MH broth growth.

Transcriptomic profiling experiments (Table S1) indicated that ribosomal proteins showed the highest level of expression, which is typically an indicator used to measure growth and metabolic activity in transcriptomic analyses (28). Similarly, both strains upregulated pathways for the utilization of several amino acids, further indicating that human milk was perceived as a suitable growth medium until bactericidal components ultimately killed the cells. Both *C. jejuni* isolates also sensed iron-limiting conditions and upregulated iron acquisition-related outer membrane receptors and transporters (ChuABCD, CfrA, and CfbpABC) and energy transduction systems (ExbB/ExbD/TonB), which is consistent with studies demonstrating that human milk is iron restricted (29) due to the reported lack of iron transporters in mammary epithelial cells (30) and the presence of lactoferrin, predicted to be an innate mechanism to hinder microbial growth by restricting biologically available iron. Although *C. jejuni* upregulated expression of ribosomal proteins, amino acid metabolism, and iron acquisition-related genes in preparation for growth in human milk, the survival studies suggested that the strains could not survive in this environment for longer periods of time. This suggests that human milk contains antimicrobial components that may kill *C. jejuni*.

### Evolution of *C. jejuni* wild-type strains in human milk and identification of inhibitory factors

**Fig 2:**
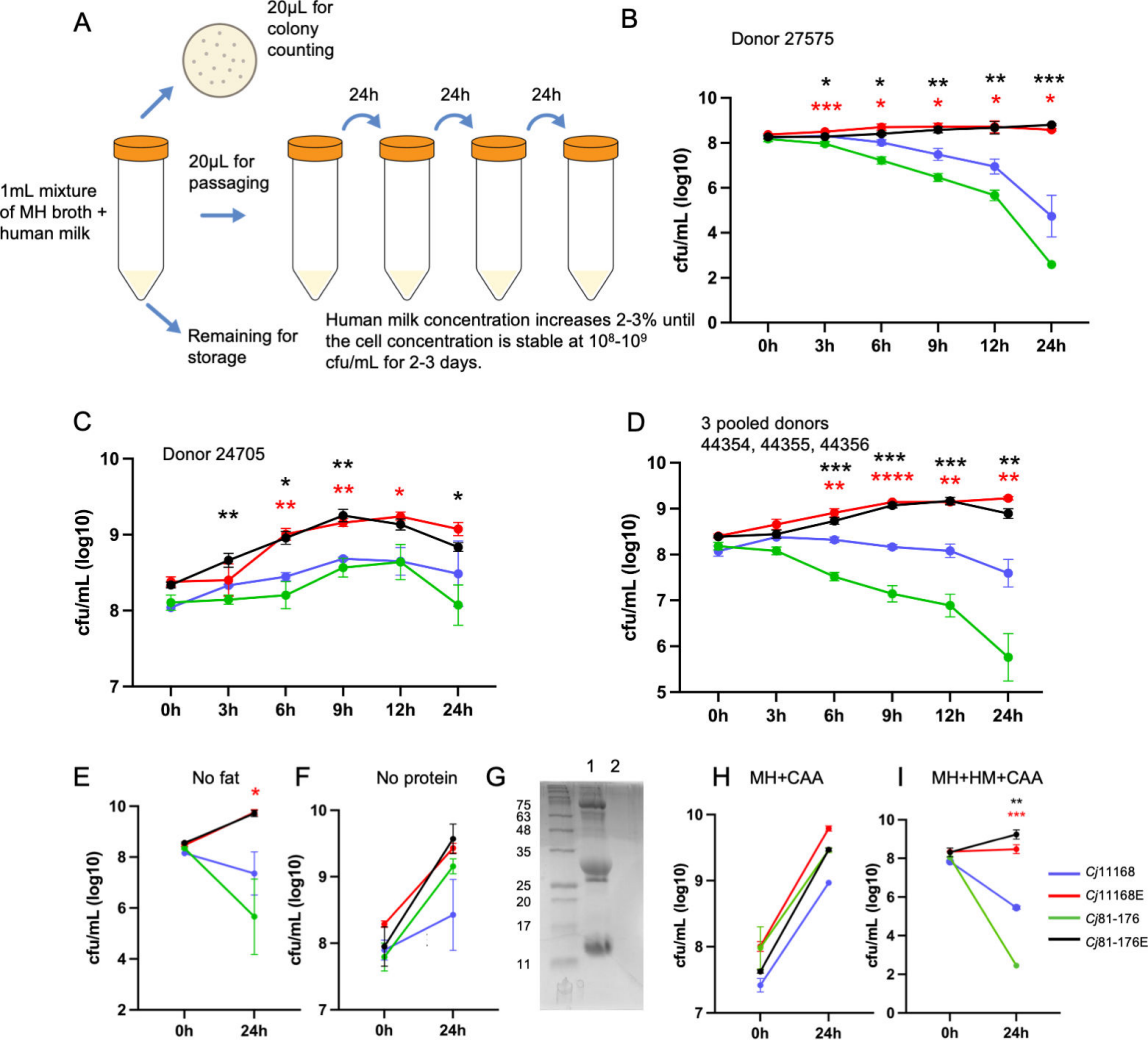
Schematic of *C. jejuni* evolution assay (**A**). Growth of *C. jejuni* in MH supplemented with human milk from donor 27595 (**B**), donor 24705 (**C**) and an equal mixture of milk from donors 44354, 44355, and 44356 (**D**). Growth of *C. jejuni* strains in MH broth supplemented with no-fat (**E**) and no-protein (**F**) human milk from donors 44354, 44355, and 44356. (**G**) SDS-PAGE of Coomassie-stained proteins from human milk from donors 44354, 44355, and 44356 (lane 1) and the proteinase K digested product (lane 2, used for panel F). (**H**) Growth of *C. jejuni* strains in MH broth supplemented with 2% casamino acids (CAAs). (**I**) Growth of *C. jejuni* strains in MH broth supplemented with human milk (equal mixture of 44354, 44355, and 44356) and 2% CAA. *C. jejuni* 11168 and *C. jejuni* 11168E were grown in 43% MH broth + 57% human milk; *C. jejuni* 81–176 and *C. jejuni* 81–176E were grown in 40% MH broth + 60% human milk, as indicated. All assays were repeated at least three times. *P < 0.05, **P < 0.01, ***P < 0.001, ****P < 0.0001 as determined by Student’s *t*-test when comparing evolved strains with their parental strains. Error bars represent the standard error of the mean.

Following our initial findings with *C. jejuni* 11168 and 81–176, we performed evolution experiments in MH broth with increasing concentrations of human milk (donor 27595) as shown in Fig. 2A. After over 100 passages, the evolved *C. jejuni* 11168 (*C. jejuni* 11168E) and 81–176 (*C. jejuni* 81–176E) strains were capable of growing in MH broth with up to 57% and 60% milk, respectively (Fig. 2B). Beyond these thresholds, we were unable to isolate viable cells. Subsequently, we repeated the growth assay in human milk from other donors (donor 24705, and using an equal mixture of donors 44354/44355/44356) and found that the evolved strains showed significantly better growth than the parental strains (Fig. 2C and D).

We next assessed whether specific human milk macromolecules were toxic to the wild-type strains. In the case of *C. jejuni* 81–176, it is well known that this strain is asaccharolytic and unable to metabolize carbohydrates, while *C. jejuni* 11168 is capable of metabolizing free fucose that is released from HMOs (22, 23, 31). When the strains were incubated in the presence of human milk, *C. jejuni* 11168 did not upregulate its L-fucose biosynthetic pathway (Table S1), although mass spectrometry analysis indicated that the majority of HMOs from each donor contained fucosylated oligosaccharides (Fig. S1; Table S2), suggesting L-fucose metabolism was not contributing to the phenotypes we were observing. In addition, the wild-type strains were unable to grow in human milk after the removal of the fat component, suggesting the protein fraction may be responsible for the antimicrobial activity (Fig. 2E). Consistent with this, both wild-type strains showed significantly improved growth in human milk after digestion with proteinase K (Fig. 2F and G). To rule out the possibility that proteinase K releases free amino acids to support the growth of *C. jejuni* in human milk, we added casamino acids into MH broth and MH broth/human milk and tested bacterial growth (Fig. 2H and I). The results show that neither *C. jejuni* 11168 nor 81–176 wild-type can grow in MH/human milk supplemented with casamino acids, indicating that the free amino acids released from proteinase K-treated proteins are not the reason why *C. jejuni* 11168 or 81–176 wild-type can grow in the presence of protease-treated human milk. The results indicate that human milk contains antimicrobial peptides and/or proteins toxic to *C. jejuni*, and proteinaceous compounds in human milk are the major contributors to *C. jejuni* growth inhibition.

### AcpP mutations impact *C. jejuni* growth in human milk

Whole-genome sequencing was performed comparing evolved strains and parental strains, and the results are summarized in Table 1. Both evolved strains showed mutations in several genes, including those encoding the major outer membrane porin, PorA, and the primary acyl carrier protein, AcpP. The amino acid changes in PorA could influence nutrients coming in and/or exclude antimicrobial compounds, such as cationic peptides, from entering since all the changes resulted in loss of the negatively charged aspartic and glutamic acid residues lining the channel (32). AcpP is a small 9,000 Da protein that acts as a coenzyme in fatty acid synthesis to carry acyl chains from one fatty acid enzyme to the next (33). Fatty acids are an important component in the bacterial cell membrane, and they are the building blocks for both phospholipids and LOS lipid A, which both impact membrane rigidity (34). These mutations may affect *C. jejuni* cell membrane functions, which further affects *C. jejuni* resistance to human milk.

**TABLE 1:** Gene mutations of *C. jejuni* 11168E and *C. jejuni* 81–176E compared with original strains

Genes	CDS position	AA change	DNA change	Protein effect	Product	Predictions
*C. jejuni* 11168E						
*acpP* (*cj0441*)	97	G33R	GGT →CGT	Substitution	Acyl carrier protein	Likely impacts LpxD and FabD interactions
*porA (cj1259*)	259	D87N	GAC → AAC	Substitution	Major outer membrane protein	Loss of negative charge in channel
*porA*	424	D142N	GAT → AAT	Substitution	Major outer membrane protein	Loss of calcium-binding site in channel
*cj0628*	511		(G)10 → (G)11	Frame shift	Putative lipoprotein	Likely loss of expression
*cj1305c*	580		(CC)4 → (CC)5	Frame shift	Hypothetical protein Cj1305c (617 family)	Likely loss of expression, mutant still motile in 81–176 with no change in glycan charge (35)
*pflA* (*cj1565c*)	1531		(T)7 → (T)6	Frame shift	Flagellar ring protein for stator anchoring	Likely loss of motility
*C. jejuni* 81–176E						
*acpP* *CJJ81176_0468*	100	A34P	GCT → CCT	Substitution	Acyl carrier protein	Likely impacts LpxD and FabD interactions
*porA* *CJJ81176_1275*	424	D142H	GAT → CAT	Substitution	Major outer membrane protein	Loss of calcium-binding site in channel
*porA*	955	E319Q	GAG → CAG	Substitution	Major outer membrane protein	Loss of negative charge in channel
*porA*	958	E320Q	GAA → CAA	Substitution	Major outer membrane protein	Loss of negative charge in channel
*purF CJJ81176_0227*	406	A136T	GCA → ACA	Substitution	Amidophosphoribosyltransferase	High-frequency changes, especially under stress, essential, so not loss of function (36)
*eptC CJJ81176_0283*	1297	G433R	GGT → CGT	Substitution	Phosphoethanolamine transferase to lipid A	Important for cationic antimicrobial peptide resistance, likely mutation has indirect effect on substrate recognition that involves H440 (37)
*pheT* *CJJ81176_0905*	614	G205A	GGT → GCT	Substitution	Phenylalanyl-tRNA synthetase, beta subunit	G to A change likely no effect on activity
*fliF* *CJJ81176_0340*	1640		(A)4 → (A)3	Frame Shift	Flagellar M-ring protein	Likely loss of motility

**Fig 3:**
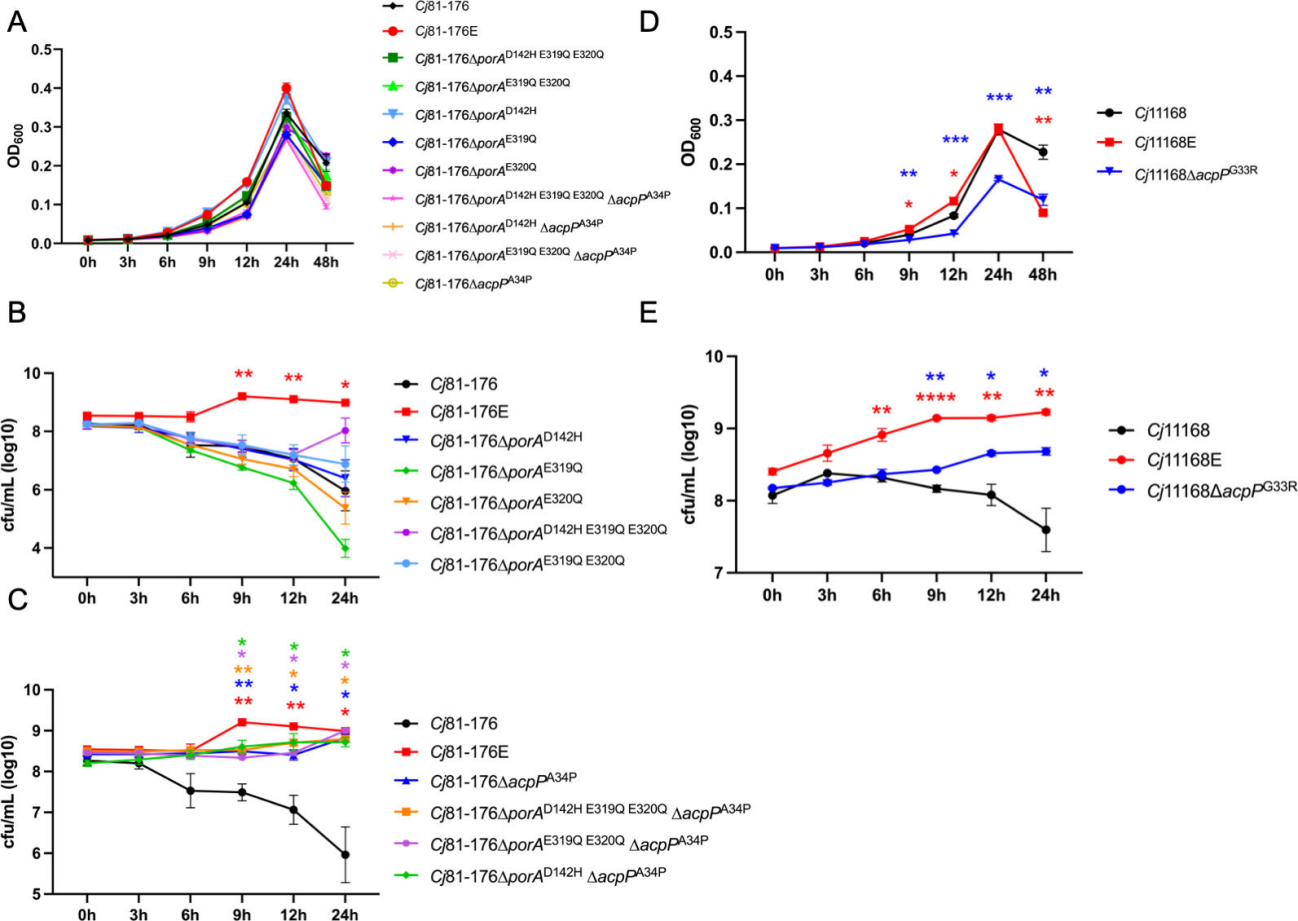
Growth of *C. jejuni acpP* and *porA* indicated point mutants in MH broth and MH broth with human milk. (**A**) The growth curve of *C. jejuni* 81–176 and its mutants in MH broth. (**B and C**) The growth curve of *C. jejuni* 81–176 and its mutants in human milk from donors 44354, 44355, and 44356. (**D**) The growth curve of *C. jejuni* 11168 and its mutants in MH broth. (**E**) The growth curve of *C. jejuni* 11168 and its mutants in human milk from donors 44354, 44355, and 44356. All assays were repeated at least three times. *P < 0.05, **P < 0.01, ***P < 0.001, ****P < 0.0001 as determined by Student’s *t*-test when comparing evolved strains with their parental strains. Error bars represent the standard error of the mean.

To investigate which changes are important for *C. jejuni* growth in human milk, we constructed several *acpP* and *porA* point mutants in *C. jejuni* 81–176 and assessed their growth profiles (Fig. 3). All constructed *C. jejuni* 81–176 mutants showed similar growth curves in MH broth (Fig. 3A). *C. jejuni* 81–176 *porA* mutants did not show significantly increased growth in the presence of human milk (Fig. 3B), while *C. jejuni* 81–176∆*acpP*^A34P^ showed significantly improved growth, although with a longer growth lag relative to *C. jejuni* 81–176E (Fig. 3C). All *acpP* and *porA* double mutants showed growth in the presence of human milk, but we did not note any synergy when both genes were mutated (Fig. 3C).

Since the *porA* mutations in 81–176 did not augment bacterial growth compared to the single mutation in *acpP*, we constructed *C. jejuni* 11168∆*acpP*^G33R^ and tested its growth in the presence of human milk. *C. jejuni* 11168∆*acpP*^G33R^ showed slower growth when compared with the wild type in MH broth (Fig. 3D), but *C. jejuni* 11168∆*acpP*^G33R^ exhibited significantly better growth in human milk compared to the wild type (Fig. 3E). Since the *acpP* mutations in both *C. jejuni* strains improved growth in human milk, we next investigated whether alterations in cell membrane architecture and acyl chain composition may be involved in *C. jejuni* resistance to human milk.

### *C. jejuni* evolution in human milk leads to cell membrane changes

**Fig 4:**
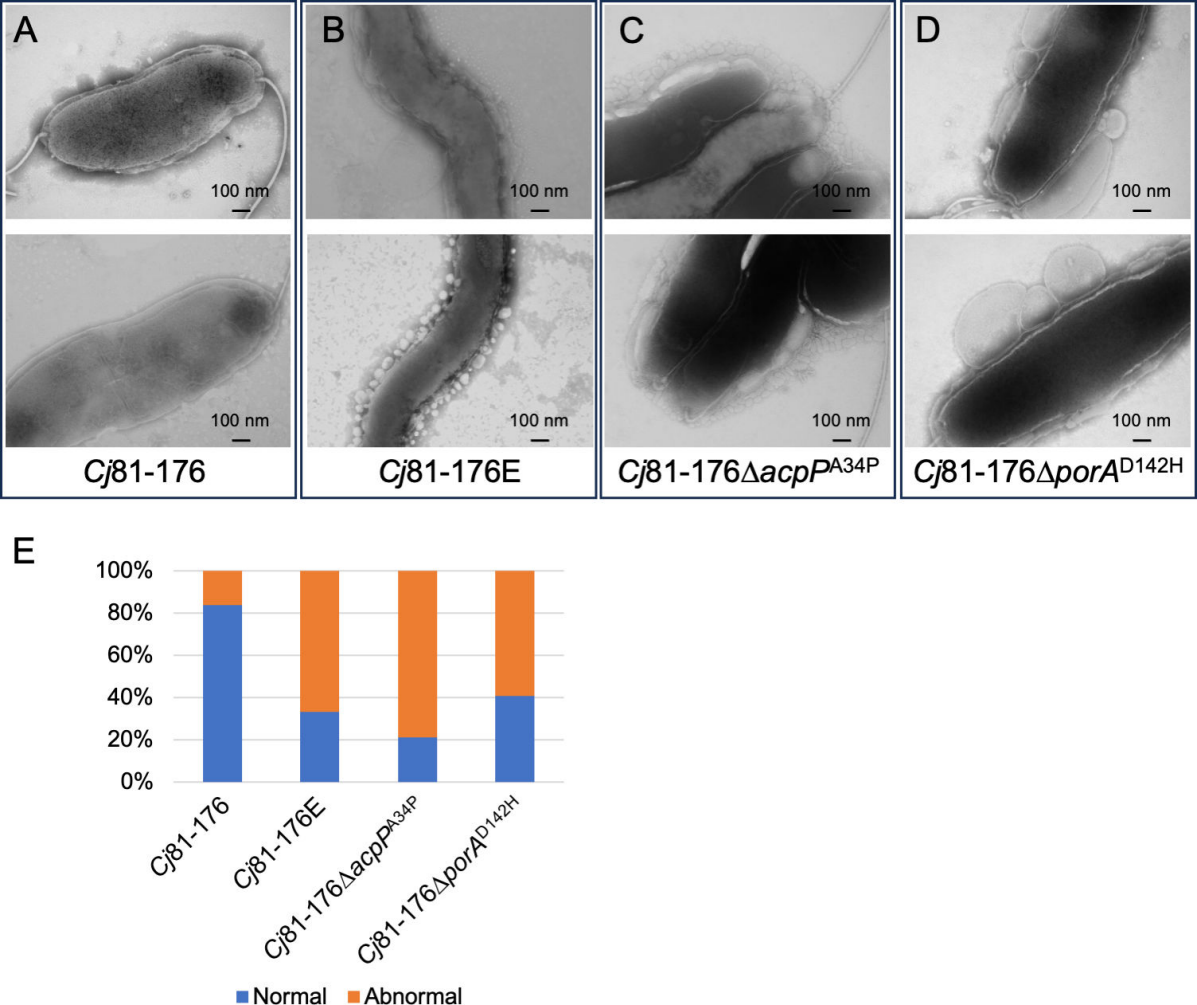
Cell morphology of *C. jejuni* 81–176 (**A**), *C. jejuni* 81–176E (**B**), *C. jejuni* 81–176∆*acpP*^A34P^ (**C**), and *C. jejuni* 81–176∆*porA*^D142H^ (**D**) observed by transmission electron microscopy (TEM) at ×20,000. (**E**) The proportion of normal and abnormal membranes observed on a grid (*n* = 50–60 cells) of *C. jejuni* 81–176, *C. jejuni* 81–176E, *C. jejuni* 81–176∆*acpP*^A34P^, and *C. jejuni* 81–176∆*porA*^D142H^ observed by TEM.

To examine cell morphology, we characterized *C. jejuni* 81–176WT, *C. jejuni* 81–176E, and *C. jejuni* 81–176∆*acpP*^A34P^ using transmission electron microscopy (TEM), including *C. jejuni* 81–176∆*porA*^D142H^ for comparison (Fig. 4).

*C. jejuni* 81–176 exhibited typical spiral-shaped morphology with smooth cell membranes (Fig. 4A), while *C. jejuni* 81–176E, *C. jejuni* 81–176∆*acpP*^A34P^, and *C. jejuni* 81–176∆*porA*^D142H^ exhibited irregular outer membranes (Fig. 4B through D). *C. jejuni* 11168∆*acpP*^G33R^ also showed a similar phenotype as *C. jejuni* 81–176∆*acpP*^A34P^ (Fig. S2). The TEM indicated distinct differences in the outer membranes of *C. jejuni* 81–176∆*acpP*^A34P^ (continuous layer of small outer membrane vesicles surrounding the cell) and *C. jejuni* 81–176∆*porA*^D142H^ (large membrane blebs protruding from the cell), with both morphologies observed in the evolved strain. Enumeration of normal and abnormal cell morphologies (Fig. 4E) indicated that most cells of *C. jejuni* 81–176E, *C. jejuni* 81–176∆*acpP*^A34P^, and *C. jejuni* 81–176∆*porA*^D142H^ exhibited abnormal cell morphology compared with wild type, pointing toward an altered membrane composition.

### *C. jejuni* lipooligosaccharides and lipid analyses

**Fig 5:**
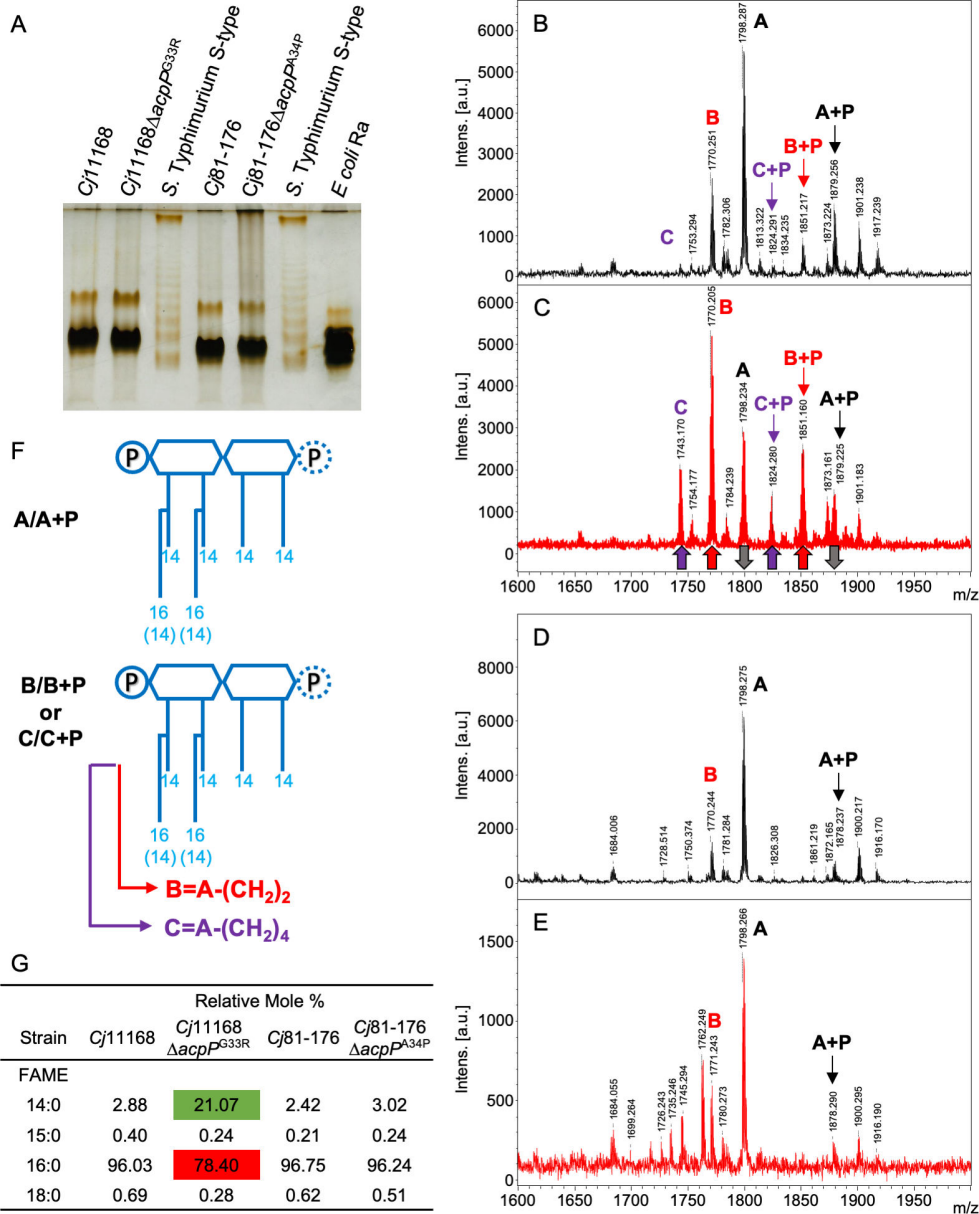
Comparative DOC-PAGE silver-stain analysis of LOS extracted from *C. jejuni* 11168 and *C. jejuni* 11168∆*acpP*^G33R^; *C. jejuni* 81–176, and *C. jejuni* 81–176∆*acpP*^A34P^ (**A**). Standards of *Salmonella enterica* ser. Typhimurium S-type LPS and *Escherichia coli* Ra-type LPS were used as molecular weight references. Mass spectrometry comparison of purified LOS from *C. jejuni* 11168 (**B**), *C. jejuni* 11168∆*acpP*^G33R^ (**C**), *C jejuni* 81–176 (**D**), and *C. jejuni* 81–176∆*acpP*^A34P^ (**E**). Arrows in panel** C** indicate LOS core components that increased or decreased in *C. jejuni* 11168∆*acpP*^G33R^. Schematic structure of components A/A + P, B/B + P, and C/C + P (**F**). (**G**) Table shows relative mole percent of ester-linked fatty acids identified by GC-MS analysis of lipid A of *C. jejuni* 11168 and 81–176 parent and *acpP* mutants (spectra in Fig. S4). Green highlights show increased 14:0 fatty acids; red shows decreased 16:00 fatty acids in 11168∆*acpP*^G33R^. FAME, fatty acid methyl ester.

Since the TEM results showed cell membrane differences, we next investigated the lipid compositions in the lipooligosaccharides (LOS) and phospholipids. *C. jejuni* 11168, *C. jejuni* 11168∆*acpP*^G33R^, *C. jejuni* 81–176, and *C. jejuni* 81–176∆*acpP*^A34P^ LOS were extracted and visualized by deoxycholic acid (DOC)-PAGE (Fig. 5A). No major changes in the electrophoretic mobility of LOS were detected between the wild-type and *acpP* mutants, suggesting no major change in the LOS oligosaccharides. Lipid A was released from LOS by mild hydrolysis, and the acylation profiles were examined by matrix-assisted laser desorption/ionization time-of-flight mass spectrometry (MALDI TOF-MS) (Fig. 5B through E, and full spectra in Fig. S3). Subsequently, the fatty acids were released from the lipid A by strong hydrolysis, converted to fatty acid methyl esters (FAME), and their composition was investigated by gas chromatography-mass spectrometry (GC-MS) (Fig. S4). MALDI TOF-MS of the *C. jejuni* 11168 lipid A consisted mainly of *mono*-and *bis-*phosphoryl hexa-acyl components (A and A + P), with the lipid A substituted with four 14:0(3-OH) acyl- and two 16:0 acyl-oxy-acyl chains (Fig. 5F). In addition, we observed a minor amount of *mono*-and *bis-*phosphoryl hexa-acyl components (B and B + P, respectively) with one of the 16:0 replaced with 14:0 and trace amounts of *mono*-and *bis-*phosphoryl hexa-acyl components (C and C + P, respectively) with two of the 16:0 acyl chains replaced with 14:0. In contrast to 11168, the 11168∆*acpP*^G33R^ lipid A contained *mono*-and *bis-*phosphoryl hexa-acyl components enriched with shorter fatty acid chains (more B and C, less A) and more *bis*-phosphorylated lipid A with shorter fatty acid chains (more components B + P and C + P) than wild type (Fig. 5G). However, we did not observe this change in acyl chain length between *C. jejuni* 81–176 and *C. jejuni* 81–176∆*acpP*^A34P^, and both strains predominantly expressed monophosphoryl lipid A (Fig. 5G).

**Fig 6:**
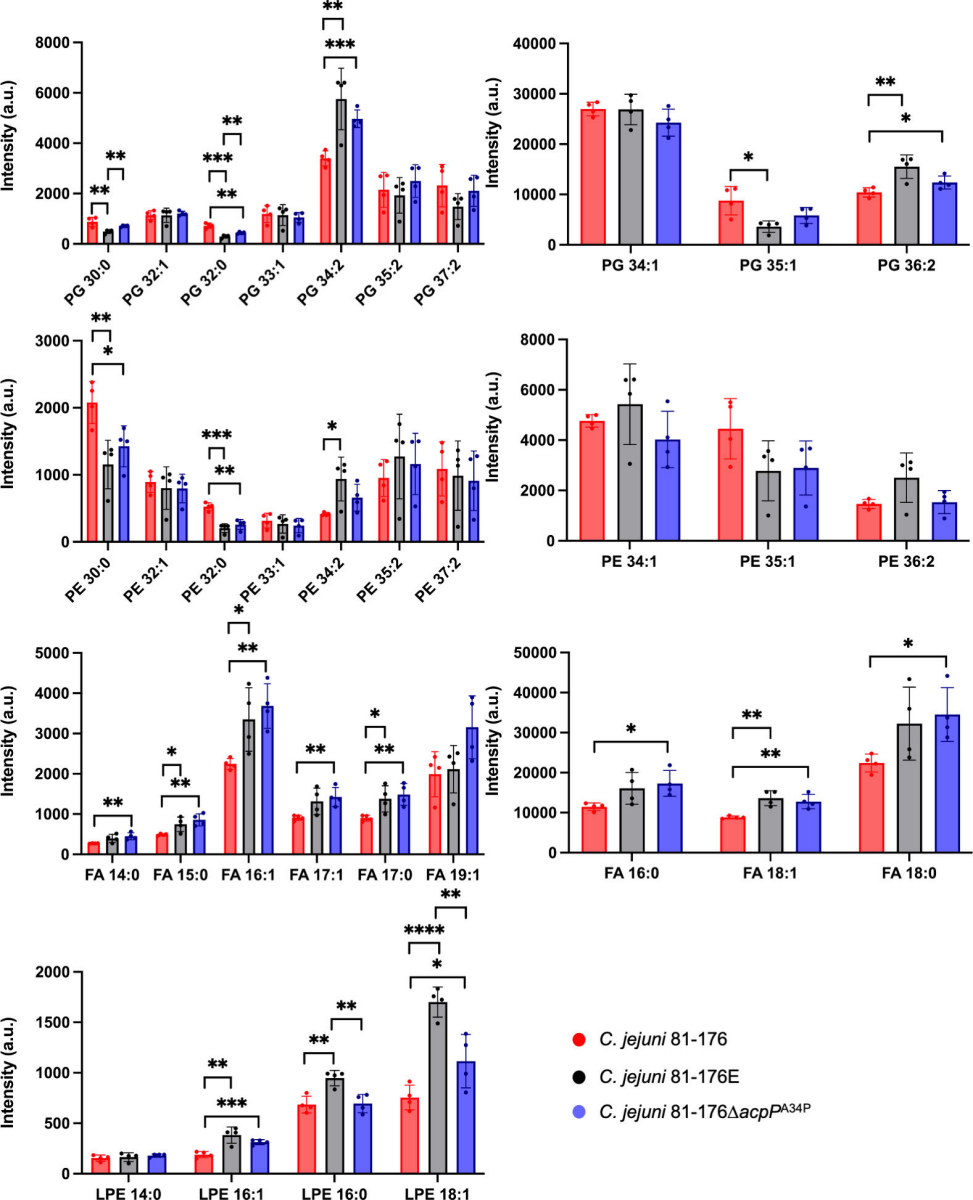
Lipid composition determined by liquid chromatography-mass spectrometry of *C. jejuni* 81–176, *C. jejuni* 81–176E, and *C. jejuni* 81–176∆*acpP*^A34P^. Error bars represent the standard deviation from quadruplicate samples. *P < 0.05, **P < 0.01, ***P < 0.001, ****P < 0.0001 as determined by Student’s *t*-test (two-tailed, two-sample equal variance) comparisons between the three samples. Data spreadsheets and comparisons with 81–176∆*porA*^D142H^ can be found in the supplemental material. FA, fatty acid; PE, phosphatidylethanolamine; PG, phosphatidylglycerol; LPE, lysophosphatidylethanolamine.

As we did not observe any differences in acyl chain length in *C. jejuni* 81–176 and 81–176∆*acpP*^A34P^, we isolated the phospholipids and fatty acids from *C. jejuni* 81–176, *C. jejuni* 81–176E, and *C. jejuni* 81–176∆*acpP*^A34P^ and analyzed their composition using liquid chromatography (LC)-mass spectrometry (MS) (Fig. 6; Table S3). *C. jejuni* 81–176∆*porA*^D142H^ and *C. jejuni* 11168 strains were also analyzed and are shown in Fig. S5 and S6, Table S3, and Table S4 but not discussed in detail here. The major cell lipids in 81–176 were phosphatidylglycerol (PG), phosphatidylethanolamine (PE), FAs, and lysophosphatidylethanolamine (LPE). The results showed similar trends for 81–176E and 81–176∆*acpP*^A34P^ compared to wild type. This included a significant increase in PG with longer and unsaturated acyl chains, with a corresponding reduction in PG with shorter acyl chains. There were also significant decreases in PE 30:0 and 32:0 in 81–176E and 81–176∆*acpP*^A34P^, together with significant increases in LPE 16:1, 16:0, and 18:1. In contrast, all detected fatty acids showed increasing trends or statistically higher levels compared to wild-type 81–176. Of interest, *C. jejuni* 81–176∆*porA*^D142H^ showed lipid compositions similar to the wild type for most lipid species, with the exception of a significant drop in all LPE levels (Fig. S5). Together, the LOS differences in *C. jejuni* 11168 and the membrane lipid differences in 81–176 provide a starting point to further investigate the link to the observed membrane morphologies and their relevance in milk resistance.

### Cell membrane function assays

**Fig 7:**
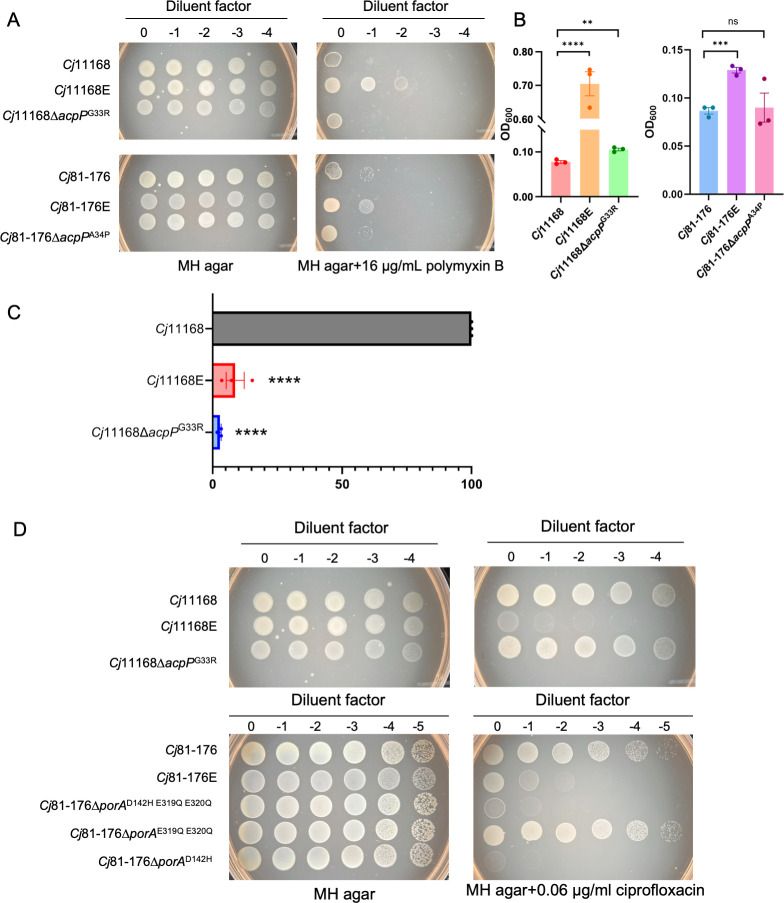
Growth of *C. jejuni* 11168, *C. jejuni* 11168E, *C. jejuni* 11168∆*acpP*^G33R^, *C. jejuni* 81–176, *C. jejuni* 81–176E, and *C. jejuni* 81–176∆*acpP*^A34P^ on MH agar plates supplemented with 16 µg/mL polymyxin (**A**). Quantitation of growth of these strains using MH broth supplemented with 8 µg/mL polymyxin B (**B**). All assays were repeated at least three times. ***P < 0.001, ****P < 0.0001 as determined by the Student’s *t*-test. Percent efficiency of plating of phage NCTC 12673 on *C. jejuni* 11168, *C. jejuni* 11168E, and *C. jejuni* 11168∆*acpP*^G33R^ (**C**). Growth of *C. jejuni* 11168, *C. jejuni* 11168E, *C. jejuni* 11168∆*acpP*^G33R^, *C. jejuni* 81–176, *C. jejuni* 81–176E, *C. jejuni* 81–176∆*porA*^D142H E319Q E320Q^*, C. jejuni* 81–176∆*porA*^E319Q E320Q^, and *C. jejuni* 81–176 *porA*^D142H^ on MH agar supplemented with 0.06 µg/mL ciprofloxacin (**D**). The *C. jejuni* 11168 comparisons in panels **A** and **D** were done on the same day, so the same MH agar plate is shown next to the antibiotic plate for ease of comparison.

We next sought to determine whether the changes in cell membrane structure and composition in the evolved strains and *acpP* mutants provide additional benefits to the microbes. Polymyxin B is a cationic detergent antibiotic targeting cell membranes by binding to lipid A and inserting into the membrane (38, 39). When growing *C. jejuni* strains on MH agar supplemented with 16 µg/mL polymyxin B (Fig. 7A) or MH broth supplemented with 8 µg/mL polymyxin B (Fig. 7B), the results indicate that both evolved strains grow significantly better than wild type. In MH broth (Fig. 7B), *C. jejuni* 11168∆*acpP*^G33R^ strain also showed significantly increased resistance to polymyxin relative to wild type, while *C. jejuni* 81–176∆*acpP*^A34P^ sensitivity did not change. These results are consistent with our observed changes in the lipid A composition of 11168∆*acpP*^G33R^ but not in 81–176∆*acpP*^A34P^ when compared with their corresponding wild-type strains.

*C. jejuni* bacteriophage NCTC 12673 is a lytic T4-like myovirus that is capable of killing *C. jejuni* 11168, while 81–176 is not susceptible to this phage (40, 41) or any *C. jejuni* phage isolated to date. It is reported that the capsular polysaccharides (CPS) are required for adsorption onto the host (42), and this is presumably through the glycolipid linker (43). To examine whether the membrane changes influence infectivity, phage plaquing assays were done with the *C. jejuni* 11168 strains (Fig. 7C) and showed that *C. jejuni* 11168E and *C. jejuni* 11168∆*acpP*^G33R^ were more resistant to NCTC 12673 infection compared to wild type.

Genome sequencing of both evolved strains showed nucleotide changes in *porA* leading to an amino acid change at D142. This aspartic acid residue is responsible for binding Ca^2+^ in the constriction zone within the channel and is necessary to prevent transport of antimicrobials such as ciprofloxacin through the porin (32). We performed ciprofloxacin sensitivity assays on *C. jejuni* 11168, 11168E, and 11168∆*acpP*^G33R^. Only *C. jejuni* 11168E, which has the PorA D142 mutation, showed increased sensitivity to ciprofloxacin, and the *acpP* mutation was not involved (Fig. 7D). We next performed the assay on *C. jejuni* 81–176 and its *porA* mutants. The results again showed that only mutants with the PorA D142 mutation showed increased sensitivity to ciprofloxacin (Fig. 7D).

The results indicate that the observed cell membrane changes not only improve *C. jejuni* survival in the presence of human milk but also significantly alter its sensitivity to antibiotics and ability to resist bacteriophage infectivity.

## DISCUSSION

Human milk is an important source of nutrients for infants, and many compounds in milk are bioactive and antimicrobial. Paradoxically, Bian and colleagues showed significantly higher levels of *Campylobacter* in the gut of breastfed infants from LMICs, and other studies have suggested that breastfeeding is a risk factor for *Campylobacter* infection in infants (24, 25). This study investigated the effect of human milk on *C. jejuni* by analyzing gene expression changes upon exposure to human milk, evolving *C. jejuni* strains to grow within human milk, and examining the role of these mutations in this process.

**Fig 8:**
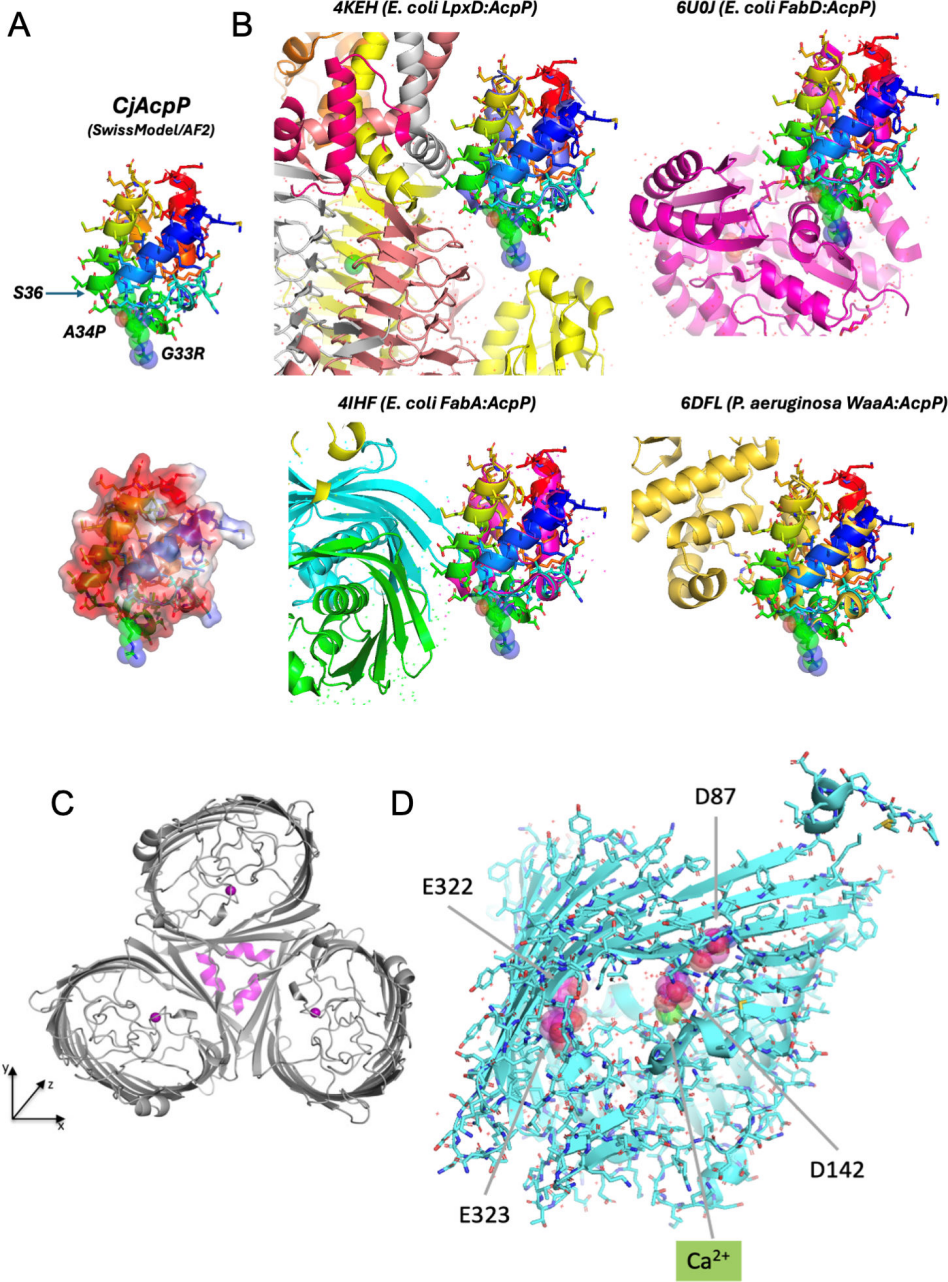
Models of observed differences in AcpP and PorA in the evolved strains. Homology model of *C. jejuni* (*Cj*) AcpP generated using the SWISS-MODEL (50) shown in ribbon and semi-transparent accessible surface representations (colored by electrostatic potential), illustrating the location of the S36 site for the 4′-phosphopantetheine prosthetic group and the two sites altered in *C. jejuni* strains 11168 and 81–176 (**A**). Views of crystal structures of four protein complexes formed with AcpP drawn from the same orientation of AcpP to help indicate some of the potential effects of mutations observed in AcpP proteins and their interactions with LpxD (N-acyltransferase to glucosamine moiety of lipid A), FabD (malonyl-CoA transacylase catalyzes the first committed step in initiation of fatty acid biosynthesis through transfer of the malonyl group from malonyl-CoA to AcpP), and other protein partners (**B**). Crystal structure of the PorA trimer (32) showing bound calcium in magenta (**C**). SWISS-MODEL was used to visualize the effects of the four amino acid substitutions observed in PorA (highlighted in space-filling representation in magenta) all within the negatively charged porin channel (**D**). The calcium ion is shown in green.

Our experiments suggest that specific amino acid changes in AcpP are important in supporting *C. jejuni* growth in human milk. AcpP is an acyl carrier protein with several distinct interaction partners, but all contribute acyl chains for phospholipid or LOS lipid A biosynthesis (33). Modeling the non-conserved amino acid changes (G33R in *C. jejuni* 11168 and A34P in 81–176) observed in the evolved strains indicates that both are located adjacent to S36, the 4′-phosphopantetheine prosthetic group binding site that interacts with the substrate (Fig. 8A). These changes do not appear to impact certain interaction partners such as FabA or WaaA, but may alter interactions with other proteins such as LpxD (N-acyltransferase to glucosamine moiety of lipid A) and FabD (malonyl-CoA transacylase catalyzing first committed step in initiation of fatty acid biosynthesis) known to be involved in lipid A and phospholipid biosynthesis, respectively (Fig. 8B) (44–46). This will be interesting to investigate further since acyl chains of these molecules are unusual in *C. jejuni*. Recent lipidomics studies examining the phospholipid composition of *C. jejuni* strain 81116 revealed the presence of an abnormally large proportion of lysophospholipids (47). Similarly, in contrast to *Escherichia coli*, *C. jejuni* is capable of synthesizing a lipid A backbone composed of GlcN3N and GlcN, attaching two N-acyl chains, followed by one O- and one N-linked acyl chain on GlcN. Subsequently, *C. jejuni* adds two longer C_16:0_ acyl chains that are ester-linked (48) to a fraction of LOS, resulting in both tetra- and hexa-acylated LOS. We detected shorter acyl chains in 11168∆*acpP*^G33R^, and this might be due to altered activity in the N-acyltransferase or changes in other acyl additions that are influenced by mutation of LpxD. It has been shown that hypoacylation of *C. jejuni* LOS is more important than the longer chain length in contributing to the dampened TLR-4-mediated inflammatory responses observed for this microbe (49). However, the shorter LOS acyl chains observed in this study could also enhance the immunogenicity of the *C. jejuni* LOS, which may influence GBS development. TLR-4 responses are also impacted by replacement of the lipid A phosphate groups with phosphoethanolamine (pEtN) (49). Interestingly, in strain 81–176, the evolved version showed an amino acid change (G433R) in EptC, the pEtN transferase to lipid A (and mobilized colistin resistance [MCR-1] homolog), which not only is thought to influence interactions with TLR-4, but is also known to provide resistance against antimicrobials such as polymyxin B (37). Although we did not detect noticeable amounts of pEtN modification in our MS studies, more analyses are required before we can eliminate this possibility.

Polymyxin B interacts with the LPS of gram-negative bacteria, and its polycationic peptide ring displaces the Ca^2+^ and Mg^2+^ bridges that stabilize the LPS through binding to the outer membrane. Fatty acid chains are important components in LPS and contribute to the insertion of polymyxin B into the outer cell membrane, leading to cell membrane disruption and cell death (51). The resistance to polymyxin B in both evolved strains suggests changes in either their LOS (as mentioned), or membrane composition, or both. The microscopy images confirmed that there were dramatic changes in membrane architecture, and MS studies of purified *C. jejuni* LOS and lipids revealed significant differences in lipid A structure in 11168 (reduced acyl chain length and increased phosphate modification) as well as lipid composition in 81–176 (increased PG acyl chain lengths, decreased levels of PE with increasing levels of LPE, and an overall increase in all free fatty acids). More detailed enzymatic and analytical studies are needed to better understand how the *C. jejuni* mutations in *acpP* and the evolved strains influence cell membrane architecture and human milk resistance.

Transmission electron microscopy images indicated there was an abundance of small outer membrane vesicles surrounding both *C. jejuni* evolved strains that was recapitulated in the *acpP* point mutants of 81–176 (Fig. 4C) and 11168 (Fig S2). Our current hypothesis is that these vesicles prevent cell membrane disruption by the unidentified AMP(s) in human milk. To further test the idea that the vesicles can act as bacterial barriers, we infected our *C. jejuni* 11168 strains with phage NCTC 12673. Both *C. jejuni* 11168E and *C. jejuni* 11168∆*acpP*^G33R^ showed significantly increased resistance to NCTC 12673 phage infection. It is known that the primary receptor for this phage is the capsular polysaccharide (42). Thus, one obvious test to be performed will be to assess the amount of CPS produced by the strains using whole-cell magic angle spinning nuclear magnetic resonance (NMR) spectroscopy (52). An alternative explanation, since we currently do not have any evidence for altered expression of CPS, is that the surrounding vesicles act as decoys to capture injected phage DNA, resulting in an abortive infection. We are currently determining the contribution of the membrane vesicles to the observed phenotypes.

Multiple amino acid substitutions were also observed in PorA from both evolved *C. jejuni* strains, and these mutations not only went beyond impacting the portal of entry for nutrients and toxic compounds, but also influenced the membrane architecture and lipid composition of the cell. This is intriguing since sequence changes in PorA have also been linked to host tropism and disease outcome, most recently being solely responsible for converting a clonal lineage of *C. jejuni* diarrheagenic isolates to abortion isolates (53). The PorA amino acid changes in our study were confined to the porin constriction zone and eliminated negatively charged residues (D/E), including D142 that is solely responsible for binding a single Ca^2+^ in the middle of the pore, leading to a narrowing in the channel (Fig. 8C and D) (32). Both *C. jejuni* 11168E and 81–176E independently evolved mutations at this Ca^2+^ binding site, and we confirmed the PorA D142 mutation can impact ciprofloxacin sensitivity as previously reported (32). We postulated that the D142 mutation could impact entry of small cationic antimicrobial peptides, but construction of this PorA mutant, along with the double and triple point mutations observed in 81–176, did not improve *C. jejuni* survival in human milk and suggested that PorA plays a minimal role in milk resistance under the conditions examined (Fig. 3). It is currently puzzling to us why both *C. jejuni* evolved strains acquired the D142 mutation in *porA* when the single, double, and triple *porA* mutations do not significantly contribute to *C. jejuni* growth in human milk and requires further study.

Besides the AcpP and PorA changes, both evolved strains showed mutations in flagella-related genes, *pflA* and *fliF*, and both evolved strains lost motility (Fig. S6). This is consistent with another study by Sher and colleagues demonstrating that loss of motility is a consequence of laboratory evolution (54). However, the studies described here do not examine the importance of these and other changes observed in our evolved strains.

In this study, we determined that the compounds inhibiting *C. jejuni* growth are proteinaceous, although lipids or other components, such as microbial bacteriocins, may have synergistic roles. We also tested the activity of the common human milk antimicrobial known as LL-37 on both evolved and wild-type strains. LL-37 was of particular interest since it is reported to enter bacterial cells and specifically bind to AcpP (11). However, this antimicrobial peptide showed similar killing of both wild-type and evolved strains (Table S5), so human milk fractionation will be needed to determine the precise identity of the proteinaceous compound(s) capable of inhibiting *C. jejuni* growth in expressed human milk unaltered by intestinal digestion.

In summary, this simpler *in vitro* study provides many insights into the potential strategies *C. jejuni* uses to combat human milk stress. We predict that the gut environment will be even more complicated since interactions between the infant and the resident gut microbiome will also impact *C. jejuni* access to nutrients and colonization potential. Future studies will assess the ability of several of the key *C. jejuni* mutants to colonize *in vivo* models and particularly examine how human milk affects *C. jejuni* intestinal colonization in a mouse model mimicking infant EED.

## MATERIALS AND METHODS

### Strains, plasmids, and growth conditions

*C. jejuni* strains were grown on MH agar plates (Criterion) or Brain Heart Infusion (BHI) agar plates (Criterion), and in MH or BHI broth at 37°C under microaerobic conditions (85% N_2_, 10% CO_2_, and 5% O_2_). *E. coli* was grown on Luria-Bertani (LB, Criterion) agar plates or in LB broth at 37°C under aerobic conditions. If required, antibiotics were added to a final concentration of 50 µg/mL for kanamycin and 25 µg/mL for chloramphenicol. All strains, plasmids, and oligonucleotides used in this study are listed in Tables S6 to S8.

### *C. jejuni* 11168 and 81–176 evolution in pasteurized human milk

Human milk was purchased from Innovative Research Inc. We pasteurized human milk at 72°C for 10 min. The overall strategy is presented in Fig. 2A. On day 1, *C. jejuni* strains were mixed with 1 mL MH broth supplemented with 1% pasteurized human milk with starting OD_600_ = 0.05. The cell culture was incubated at 37°C under microaerobic conditions at 150 rpm for 24 h. On the next day, 20 µL cell culture was transferred to a new human milk and MH broth mixture with the same concentration as the previous day. The cell culture was incubated under the same conditions, and another 20 µL culture was diluted serially and plated for colony counting, while the remainder of the culture was mixed with 300 µL 80% glycerol to store at −80°C. When the *C. jejuni* colony counts were stable at approximately 10^8^–10^9^ CFU/mL for 2–3 days, the human milk concentration was increased by 2%–3%, and the same steps were repeated until the human milk concentration could not be increased further.

### *C. jejuni* growth curve in human milk and MH broth

*C. jejuni* strains were harvested in MH broth and incubated with 1 mL of MH broth and pasteurized human milk at a starting OD_600_ = 0.05. The cell cultures were incubated at 37°C under microaerobic conditions with shaking at 150 rpm. Every 3 h, 10 µL cell culture was taken out for serial dilution and colony counting.

To perform *C. jejuni* growth curves in MH broth, *C. jejuni* cells were grown in 5 mL MH broth in 50 mL Falcon tubes with a starting OD_600_ = 0.05 in semi-micro cuvette and incubated at 150 rpm under microaerobic conditions at 37°C. At each time point, 100 µL cell culture was added into 96-well plates in each well to measure OD_600_.

### *C. jejuni* whole-genome extraction and sequencing

*C. jejuni* DNA was extracted as described (55). Whole-genome sequencing of *C. jejuni* was performed using libraries prepared with the Illumina DNA Prep Tagmentation kit (Illumina, San Diego, CA) and sequencing on an Illumina MiSeq instrument as described previously (56). Illumina paired sequence reads from each sample were mapped to the appropriate reference *C. jejuni* genome, *C. jejuni* strain 81–176 genome (NC_008787), or *C. jejuni* strain NCTC 11168 (AL111168) using Geneious Mapper with low-sensitivity settings (<10% mismatch between read and reference) and at least 20× coverage and 80% of reads representing a single nucleotide polymorphism. Short read sequence data for all the samples are available at National Center for Biotechnology Information’s Sequence Read Archive with accession numbers SRR28566029–SRR28566032.

### Construction of *C. jejuni* mutants

To construct *C. jejuni* 11168∆*acpP*^G33R^ and *C. jejuni* 81–176∆*acpP*^A34P^, pBZ136 and pBZ137 were constructed. pBZ136 and 137 were constructed by using CS-1024 and CS-1095 to amplify a 1.2 kb upstream fragment including *acpP*^G33R^ and *acpP*^A34P^ using *C. jejuni* 11168E and *C. jejuni* 81–176E as template. CS-1026 and CS-1027 were used to amplify a 1 kb downstream fragment of *acpP*; these two fragments were ligated into the vector pGEM-T-easy harboring a kanamycin resistant gene.

To construct *porA* point variants in *C. jejuni* 81–176, pBZ133, pBZ141, pBZ142, pBZ143, and pBZ156 were constructed. pBZ133 was constructed by using CS-1094 and CS-1034 to amplify *porA* from *C. jejuni* 81–176E, which contains all *porA* mutation sites. CS-1038 and CS-1048 were used to amplify a 500 bp downstream fragment of *porA* in *C. jejuni* 81–176E; these two fragments were inserted into the vector pGEM-T-easy harboring a chloramphenicol resistant gene. To construct pBZ141, CS-1094, and CS-1012, CS-1034 and CS-1013 were used to amplify the two fragments needed for PCR overlap of *porA*^D142H^, then CS-1094 and CS-1034 were used to combine the fragments above and ligated into the same vector as pBZ133 followed by insertion of the downstream fragment. Construction of the other plasmids was the same as pBZ141, but different primers were used (Table S8).

To construct the *acpP* and *porA* double mutant, pBZ141, pBZ133, and pBZ156 were electroporated into *C. jejuni* 81–176∆*acpP*^A34P^. All *C. jejuni* clones were verified by PCR and Sanger sequencing.

### Removal of lipids and protein from human milk

To remove lipids, human milk was centrifuged at 14,000 rpm for 15 min at 4°C, and the bottom layer was used for further studies. To digest protein in human milk, 1 mL human milk was mixed with 10 µL of 20 mg/mL proteinase K and incubated at 37°C overnight. Proteinase K was inactivated by heating the sample at 95°C for 10 min.

### Transmission electron microscopy

*C. jejuni* was grown in MH broth at 150 rpm under microaerobic conditions at 37°C for 18 h. Cell cultures were centrifuged at 4,700 rpm for 15 min at 4℃ and resuspended in sterile 1× phosphate-buffered saline to OD_600_ = 1. Then, 300-mesh carbon-only grids were used for this study. The preparation is the same as previously described (57). The microscopy was done on the JOEL JEM1011 TEM at the Georgia Electron Microscopy facility at the University of Georgia (JEOL Inc, Akishima, Tokyo, Japan).

### Polymyxin B and ciprofloxacin sensitivity assay

*C. jejuni* cells were harvested in MH broth, adjusted OD_600_ = 1, and serially diluted at a 1:10 ratio. Then, 10 µL cell suspensions were added onto MH agar plates supplemented with 16 µg/mL polymyxin B or MH agar alone (as control). The plates were dried until all cell suspensions were absorbed, and the plates were incubated at 37°C under microaerobic conditions for 2 days. *C. jejuni* cells were also grown in MH broth supplemented with 8 µg/mL polymyxin B at starting OD_600_ = 0.05. After 24 h incubation at 150 rpm at 37°C under microaerobic conditions, cell OD_600_ was measured. For ciprofloxacin sensitivity assay, the method was the same as the polymyxin B agar assay, except the MH agar was supplemented with 0.06 µg/mL ciprofloxacin.

### Phage plaquing assay

*C. jejuni* cells were harvested from BHI agar plates and OD_600_ was adjusted to 0.35–0.4 in BHI broth. Then, 5 mL cell suspension was added to a sterile petri dish and incubated at 37°C under microaerobic conditions for 4 h. After 4 h incubation, cell density was adjusted to 0.35–0.4, then 500 µL cell culture was mixed with 5 mL 45°C pre-warmed 0.6% BHI agar and poured on BHI agar plates. The plate was solidified for 10 min, then 10 µL serial diluted phage NCTC 12673 was added on the edge of the plate until totally absorbed. The plate was incubated at 37°C under microaerobic conditions overnight.

### Hot water-phenol LOS purification

*C. jejuni* LOS was purified as previously described (58). Coextracted phospholipids were removed from the LOS by three consecutive 90% EtOH washes and centrifugation at 3,000 × *g* for 20 min at 4°C (59).

### Analysis of fatty acids constituting lipid A

Fatty acid constituting lipid A was released after hydrolysis of the LOS with 4 N HCl for 4 h, at 100℃ followed by threefold extraction of the hydrolysate with chloroform (1:1, vol/vol) and conversion of the extracted fatty acids to methyl esters (FAMEs) and (TMS )trimethylsilyl-methyl esters (for hydroxylated fatty acids), as previously described (59). The acidic methanolysis of fatty acids was done with 1 M HCl-methanol at 80°C for 1 h. The FAMEs were analyzed by GC-MS performed on an Agilent AT 7890A GC system interfaced to a 5975B MSD, using an EC-1 fused silica capillary column (30 m × 0.25 mm ID), and the following temperature gradients: 80°C for 2 min, then ramped to 140°C at 20°C/min with a 2 min hold, and to 200°C at 2°C/min, followed by an increase to 250°C at 30°C/min with a 5 min hold.

### DOC-PAGE analysis and staining

The LOS/LPS was resolved in PAGE by using 18% acrylamide and DOC detergent (60). The PAGE was developed with a silver stain reagent kit (Bio-Rad) after oxidation with sodium periodate.

### MALDI-TOF analysis of lipid A

The LOS was dissolved in 10 mM sodium acetate buffer (pH 4.5) and incubated at 100°C for 60 min with constant stirring. The reaction mixture was placed on ice; lipid A was extracted three times from the acetate buffer with chloroform (1:1, vol/vol); and the chloroform phase was dried down. The dry lipid A was dissolved in chloroform:methanol solution (3:1, vol/vol), mixed with 0.5 M 2,4,6-trihydroxyacetophenone matrix (Sigma) in methanol in a 1:1 ratio (vol/vol), and transferred onto a MALDI plate. The spectra were acquired on a Bruker Microflex LRF MALDI-TOF MS system in negative reflector ionization mode [M-H]^−_._^

### Lipid analysis

Bacteria pellets were extracted using the Bligh and Dyer method, as described previously (61, 62). The optical densities (OD_600 nm_) were measured for each sample following resuspension and sonication of the pellets in 0.5 mL of water. A 200 µL portion of each sample was transferred to glass centrifuge tubes, to which 2 mL of chilled 1:2 chloroform:methanol was added. After vortexing on and off for 5 min, an additional 0.5 mL each of chilled water and chloroform was added to induce phase separation. The samples were briefly mixed then centrifuged for 10 min. The lower organic layer was collected into new glass tubes and dried in a speed-vac concentrator. The dried extracts were reconstituted in 1:1 chloroform:methanol. For LC-MS, an additional 4× dilution of the extracts was prepared in 1:1 chloroform:methanol. A 5 µL aliquot of the diluted extract was transferred to LC vials, dried under vacuum, and reconstituted in 200 µL hydrophilic interaction liquid chromatography (HILIC) mobile phase A.

Lipid extracts were analyzed using a Waters Acquity UPLC system equipped with a hydrophilic interaction column (Waters CORTECS HILIC, 2.1 × 100 mm, 1.6 µm) that was coupled to a Waters Synapt XS ion mobility-mass spectrometer. Mobile phase A (MPA) consisted of 95% acetonitrile/5% water with 10 mM ammonium acetate. Mobile phase B was 50% acetonitrile/50% water with 10 mM ammonium acetate. The column was held at 40°C. Lipids were separated based on headgroup polarity using a gradient from 100% MPA to 60% MPA over 7 min with a flow rate of 0.5 mL/min (63). Data were collected in negative ionization mode over 50–1,200 *m*/*z* with the following settings: capillary voltage, −2 kV; sampling cone, 80 V; source offset, 4 V; source temperature, 150°C; desolvation temperature, 500°C; desolvation gas, 1,000 L/h; and cone gas, 50 L/h. Ion mobility separation was performed with a traveling wave height of 40 V and velocity of 550 m/s. Data-independent MS/MS was collected with a collision energy ramp of 35–50 eV.

Data analysis was performed in Progenesis QI (v.3.0, Nonlinear Dynamics) with lockmass correction and normalization to OD_600_ measurement. Features that were differentially abundant in the data set were selected based on an analysis of variance with *P* ≤ 0.01 and prioritized based on PCA loadings and OPLS-DA S-plot (EZinfo v.3.0, Umetrics). Lipids were identified first by headgroup based on retention time matching against standards and then by accurate mass against an in-house database of bacteria lipid species with a tolerance of ±10 ppm. Raw data files and the aligned peak table are available on the MassIVE repository (MSV000094612, doi:10.25345/C5B853V54).

## SUPPLEMENTAL MATERIAL

Fig. S1 mbio.03997-24-s0001.pdf HMO pie charts.

Fig. S2 mbio.03997-24-s0002.pdf 11168 TEM.

Fig. S3 mbio.03997-24-s0003.pdf LC-MS.

Fig. S4 mbio.03997-24-s0004.pdf GC-MS.

Fig. S5 mbio.03997-24-s0005.pdf Lipids.

Fig. S6 mbio.03997-24-s0006.pdf Motility.

Tables S6 to S8 mbio.03997-24-s0007.pdf Strains, plasmids, and primers.

Captions mbio.03997-24-s0008.pdf Details for Tables S1-S4.

Supplemental tables mbio.03997-24-s0009.xlsx Tables S1 to S4.

Table S5 mbio.03997-24-s0010.pdf LL-37.

ASM does not own the copyrights to Supplemental Material that may be linked to, or accessed through, an article. The authors have granted ASM a non-exclusive, world-wide license to publish the Supplemental Material files. Please contact the corresponding author directly for reuse.
