# Supplementary material for: Campylobacter jejuni resistance to human milk involves the acyl carrier protein AcpP
Source: mBio. 2025 Feb 25;16(4):e03997-24. doi: 10.1128/mbio.03997-24 (PMC11980577; doi:10.1128/mbio.03997-24)
Supplement: Fig. S1 — HMO pie charts. [file mbio.03997-24-s0001.pdf]

Supplemental material – Methods and Figure 1

Campylobacter jejuni resistance to human milk involves the acyl carrier protein AcpP

Bibi Zhou[a,b], Jolene M. Garber[a,b*], James Butcher[c], Artur Muszynski[b], Rebekah L. Casey[d], Steven Huynh[e], Stephanie Archer-Hartmann[b], Sara Porfirio[b], Ashley M. Rogers[a,b], Parastoo Azadi[b], Craig T. Parker[e], Kenneth K. S. Ng[f], Kelly M. Hines[d], Alain Stintzi[c] and Christine M. Szymanski[a,b#]

[a]Department of Microbiology, University of Georgia, Athens, GA, USA.

[b]Complex Carbohydrate Research Center, University of Georgia, Athens, GA, USA.

[c]School of Pharmaceutical Sciences, Ottawa Institute of Systems Biology and Department of Biochemistry, Microbiology and Immunology, Faculty of Medicine, University of Ottawa, Ottawa, Ontario, Canada.

[d]Department of Chemistry, University of Georgia, Athens, GA, USA.

[e]Agricultural Research Service, U.S. Department of Agriculture, Produce Safety and Microbiology Research Unit, Albany, CA, USA.

[f]Department of Chemistry and Biochemistry, University of Windsor, Windsor, ON, Canada

Materials and methods

RNA extraction and analysis of *C. jejuni* incubated in human milk

RNA was extracted from cultures as described previously (1-3). Briefly, *C. jejuni* cultures were pelleted by centrifugation and total RNA was extracted using a hot-phenol extraction method and genomic DNA was removed by treating with RNase-free DNase I. The absence of genomic DNA was confirmed by PCR. Final RNA quality and quantity was ascertained using a Agilent Bioanalyzer. Strand-specific, rRNA-depleted sequencing libraries were generated and sequenced on an Illumina HiSeq 4000 by Genome Quebec.

Cutadapt was used to remove potential adapter contamination, rRNA reads were removed using SortMeRNA (4) and the reads aligned to either the 11168 (NC_002163.1) or 81-176 (ASM1552v1) genome as appropriate using bowtie2 (5) with local alignment and very-sensitive settings. Filtered and rRNA depleted sequencing reads have been deposited to the NCBI SRA archive under accession number PRJNA1104578. Reads aligning to transcriptional regions were

counted using HTSeq (6). Gene differential expression analysis was performed using DESeq2 (7) as we have previously described (2, 8, 9) with results considered significant with an absolute fold change >2 and a Benjamini and Hochberg corrected $p \leq 0.05$ (Table S1).

HMO analyses in human milk

Human milk samples were thawed on ice and an aliquot of 50 µl was taken from each sample and used for oligosaccharides isolation. The remaining sample was stored at -80°C. According to the protocol described by Totten *et al*. (10), the samples were diluted with 50 µL of nanopure water and delipidated by centrifugation at 14000 rpm, 4°C, 30 minutes. The defatted milk was subjected to ethanol precipitation (-80°C, 1 hour) to remove proteins and obtain an oligosaccharide-enriched sample. The samples (200 µg) were further purified using a C18 SPE cartridge (Discovery® DSC-18 SPE Tube 100 mg, Supelco) to remove glycolipids that may have remained after delipidation. Oligosaccharides were eluted with nanopure water. Following C18 SPE cleanup, the samples were spiked with 10 µg of xylotriose as internal standard and reduced with sodium borohydride in PBS (final concentration ~4 mg/mL) at room temperature overnight. The reduced oligosaccharides were further purified using porous graphitized carbon cartridges (Extract-Clean SPE Carbo 150 mg, GRACE Discovery Sciences) to remove salts. Oligosaccharides were eluted with 20% acetonitrile followed by 40% acetonitrile in 0.05% trifluoroacetic acid (TFA). The oligosaccharides were permethylated for structural characterization by mass spectrometry (11). Briefly, the dried eluates were methylated with NaOH and iodomethane. The reaction was quenched with water and the per-*O*-methylated carbohydrates were extracted with organic solvent. The permethylated glycans were dissolved with methanol and crystallized with α-dihydroxybenzoic acid (DHBA) matrix. Analysis of glycans present in the

samples was performed by MALDI-TOF-MS using AB SCIEX TOF/TOF 5800 (Applied Biosystem MDS Analytical Technologies).

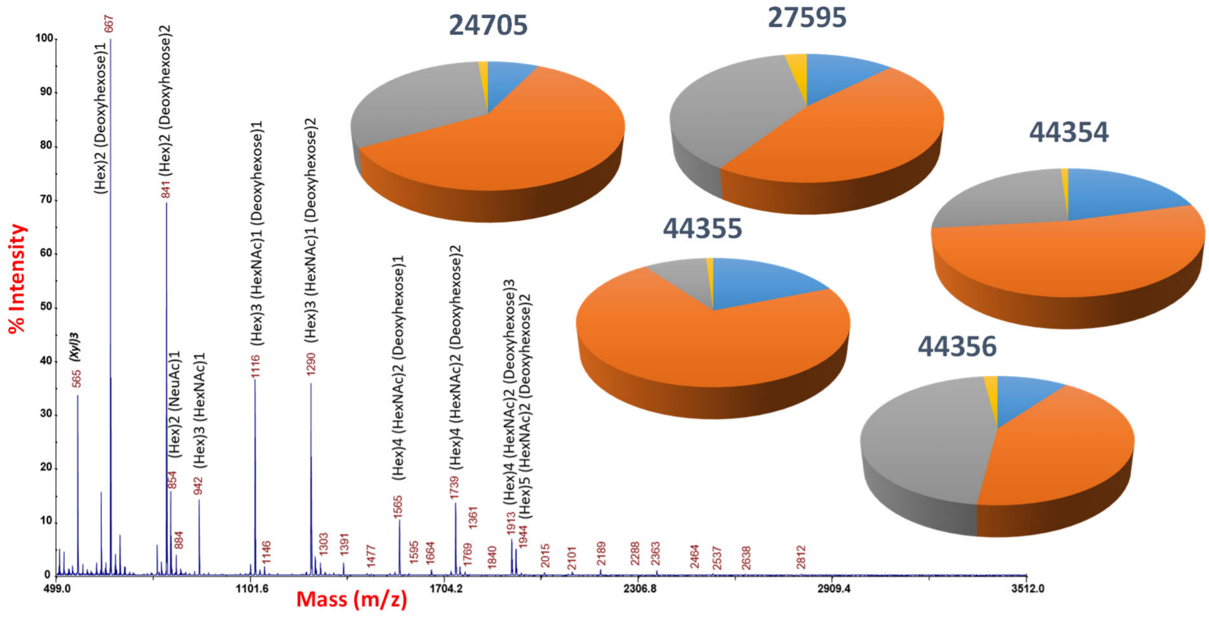

Supplemental Figure 1. MALDI-TOF-MS of reduced, permethylated HMOs from one human milk sample from donor 27595 is shown as a representative. Data from mass spectra of all human milk samples (Table S2) was compiled into pie charts to indicate proportion of HMOs in total sample modified with 0 (blue), 1 (orange), 2 (grey) or 3+ (yellow) fucose residues. Donor number is indicated above each pie chart.
