# Supplementary material for: Campylobacter jejuni resistance to human milk involves the acyl carrier protein AcpP
Source: mBio. 2025 Feb 25;16(4):e03997-24. doi: 10.1128/mbio.03997-24 (PMC11980577; doi:10.1128/mbio.03997-24)
Supplement: Fig. S5 — Lipids. [file mbio.03997-24-s0005.pdf]

Supplemental material – Figure 5

Campylobacter jejuni resistance to human milk involves the acyl carrier protein AcpP

Bibi Zhou[a,b], Jolene M. Garber[a,b*], James Butcher[c], Artur Muszynski[b], Rebekah L. Casey[d], Steven Huynh[e], Stephanie Archer-Hartmann[b], Sara Porfirio[b], Ashley M. Rogers[a,b], Parastoo Azadi[b], Craig T. Parker[e], Kenneth K. S. Ng[f], Kelly M. Hines[d], Alain Stintzi[c] and Christine M. Szymanski[a,b#]

[a]Department of Microbiology, University of Georgia, Athens, GA, USA.

[b]Complex Carbohydrate Research Center, University of Georgia, Athens, GA, USA.

[c]School of Pharmaceutical Sciences, Ottawa Institute of Systems Biology and Department of Biochemistry, Microbiology and Immunology, Faculty of Medicine, University of Ottawa, Ottawa, Ontario, Canada.

[d]Department of Chemistry, University of Georgia, Athens, GA, USA.

[e]Agricultural Research Service, U.S. Department of Agriculture, Produce Safety and Microbiology Research Unit, Albany, CA, USA.

[f]Department of Chemistry and Biochemistry, University of Windsor, Windsor, ON, Canada

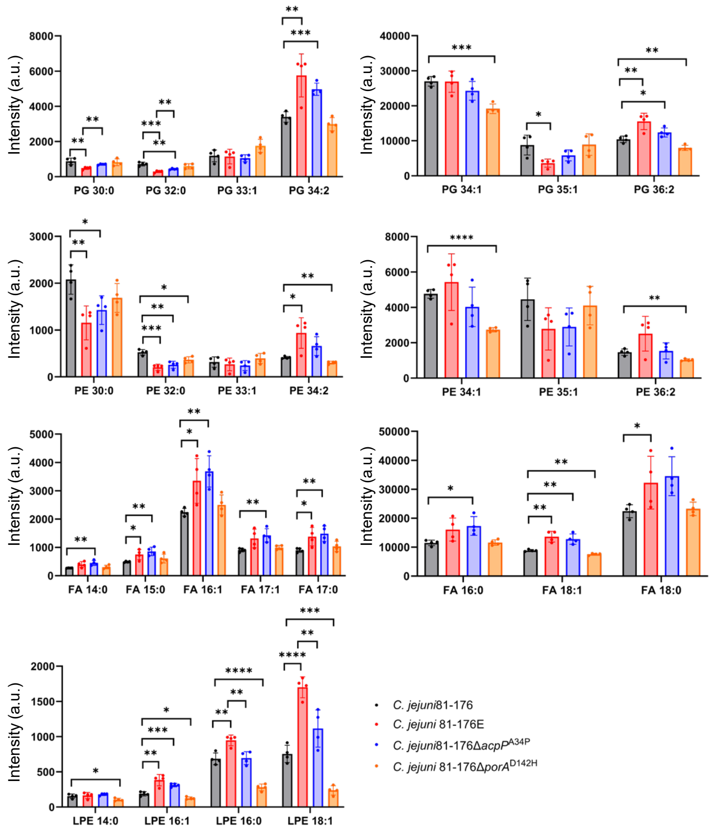

Supplementary Figure 5. Lipid composition determined by liquid chromatography-mass spectrometry from *C. jejuni* 81-176, *C. jejuni* 81-176E, *C. jejuni* 81-176Δ*acpP*^{A34P}, and *C. jejuni* 81-176Δ*porA*^{D142H}. Error bars represent the standard deviation from quadruplicate samples. PG: peptidoglycan; PE: phosphatidylethanolamine; FA: fatty acid; LPE: lyso phosphatidylethanolamine. *: $p<0.05$; **: $p<0.01$; ***: $p<0.001$; ****: $p<0.0001$ as determined by Student's t-test (two-tailed, two sample equal variance) comparisons between the three samples.
