# Supplementary material for: Campylobacter jejuni resistance to human milk involves the acyl carrier protein AcpP
Source: mBio. 2025 Feb 25;16(4):e03997-24. doi: 10.1128/mbio.03997-24 (PMC11980577; doi:10.1128/mbio.03997-24)
Supplement: Fig. S6 — Motility. [file mbio.03997-24-s0006.pdf]

Supplemental material – Methods and Figure 6

Campylobacter jejuni resistance to human milk involves the acyl carrier protein AcpP

Bibi Zhou[a,b], Jolene M. Garber[a,b*], James Butcher[c], Artur Muszynski[b], Rebekah L. Casey[d], Steven Huynh[e], Stephanie Archer-Hartmann[b], Sara Porfirio[b], Ashley M. Rogers[a,b], Parastoo Azadi[b], Craig T. Parker[e], Kenneth K. S. Ng[f], Kelly M. Hines[d], Alain Stintzi[c] and Christine M. Szymanski[a,b#]

[a]Department of Microbiology, University of Georgia, Athens, GA, USA.

[b]Complex Carbohydrate Research Center, University of Georgia, Athens, GA, USA.

[c]School of Pharmaceutical Sciences, Ottawa Institute of Systems Biology and Department of Biochemistry, Microbiology and Immunology, Faculty of Medicine, University of Ottawa, Ottawa, Ontario, Canada.

[d]Department of Chemistry, University of Georgia, Athens, GA, USA.

[e]Agricultural Research Service, U.S. Department of Agriculture, Produce Safety and Microbiology Research Unit, Albany, CA, USA.

[f]Department of Chemistry and Biochemistry, University of Windsor, Windsor, ON, Canada

Motility assay

C. jejuni cells were harvested in MH broth and OD_{600} was adjusted to 1. One microliter of the cell suspension was inserted into 0.4% MH agar plates and the plates were incubated at 37 °C under microaerobic conditions for two days. The zone of motility was then measured.

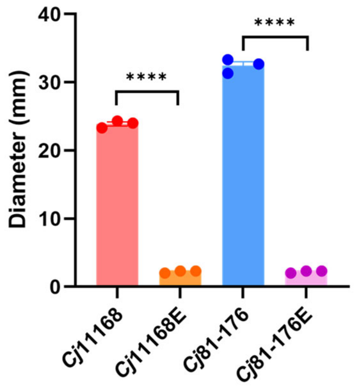

Supplementary Figure 6. The motility of *C. jejuni* 11168, *C. jejuni* 11168E, *C. jejuni* 81-176 and *C. jejuni* 81-176E determined by soft agar motility assays. All assays were repeated at least three times. ****: p<0.0001 as determined by the Student's T test.
