# Supplementary material for: Campylobacter jejuni resistance to human milk involves the acyl carrier protein AcpP
Source: mBio. 2025 Feb 25;16(4):e03997-24. doi: 10.1128/mbio.03997-24 (PMC11980577; doi:10.1128/mbio.03997-24)
Supplement: Tables S6 to S8 — Strains, plasmids, and primers. [file mbio.03997-24-s0007.pdf]

Supplemental material – Tables S6 – S8 and References

Campylobacter jejuni resistance to human milk involves the acyl carrier protein AcpP

Bibi Zhou[a,b], Jolene M. Garber[a,b*], James Butcher[c], Artur Muszynski[b], Rebekah L. Casey[d], Steven Huynh[e], Stephanie Archer-Hartmann[b], Sara Porfirio[b], Ashley M. Rogers[a,b], Parastoo Azadi[b], Craig T. Parker[e], Kenneth K. S. Ng[f], Kelly M. Hines[d], Alain Stintzi[c] and Christine M. Szymanski[a,b#]

[a]Department of Microbiology, University of Georgia, Athens, GA, USA.

[b]Complex Carbohydrate Research Center, University of Georgia, Athens, GA, USA.

[c]School of Pharmaceutical Sciences, Ottawa Institute of Systems Biology and Department of Biochemistry, Microbiology and Immunology, Faculty of Medicine, University of Ottawa, Ottawa, Ontario, Canada.

[d]Department of Chemistry, University of Georgia, Athens, GA, USA.

[e]Agricultural Research Service, U.S. Department of Agriculture, Produce Safety and Microbiology Research Unit, Albany, CA, USA.

[f]Department of Chemistry and Biochemistry, University of Windsor, Windsor, ON, Canada

Table S6. Strains used in this study.

**Strains**	**Description/genotype**	**Source**
C. jejuni 11168	Clinical isolate used for genome sequencing	(12)
C. jejuni 81-176	Clinical isolate	(13)
C. jejuni 11168 E	*C. jejuni* 11168 evolved strain from human breast milk	This study
C. jejuni 81-176 E	*C. jejuni* 81-176 evolved strain from human breast milk	This study
C. jejuni 11168Δ*acpP* ^{G33R}	*C. jejuni* 11168 *acpP* ^{G33R} point mutant, Kan^R.	This study
C. jejuni 81-176Δ*acpP* ^{A34P}	*C. jejuni* 81-176 *acpP* ^{A34P} point mutant, Kan^R.	This study
C. jejuni 81-176Δ*porA* ^{D142H}	*C. jejuni* 81-176 *porA* ^{D142H} point mutant, Cm^R.	This study
C. jejuni 81-176Δ*porA* ^{E319Q}	*C. jejuni* 81-176 *porA* ^{E319Q} point mutant, Cm^R.	This study
C. jejuni 81-176Δ*porA* ^{E320Q}	*C. jejuni* 81-176 *porA* ^{E320Q} point mutant, Cm^R.	This study
C. jejuni 81-176Δ*porA* $^{D142H\ E319Q\ E320Q}$	*C. jejuni* 81-176 *porA* $^{D142H\ E319Q\ E320Q}$ point mutant, Cm^R.	This study
C. jejuni 81-176Δ*porA* $^{E319Q\ E320Q}$	*C. jejuni* 81-176 *porA* $^{E319Q\ E320Q}$ point mutant, Cm^R.	This study
C. jejuni 81-176Δ*porA* $^{D142H\ E319Q\ E320Q}$ Δ*acpP* ^{A34P}	*C. jejuni* 81-176 *porA* $^{D142H\ E319Q\ E320Q}$ and *acpP* ^{A34P} point mutant, Kan^R, Cm^R.	This study
C. jejuni 81-176Δ*porA* $^{E319Q\ E320Q}$ Δ*acpP* ^{A34P}	*C. jejuni* 81-176 *porA* $^{E319Q\ E320Q}$ and *acpP* ^{A34P} point mutant, Kan^R, Cm^R.	This study
C. jejuni 81-176Δ*porA* ^{D142H} Δ*acpP* ^{A34P}	*C. jejuni* 81-176 *porA* ^{D142H} and *acpP* ^{A34P} point mutant, Kan^R, Cm^R.	This study
E. coli Top10	F- *mcrA (mrr-hsdRMS-mcrBC) 80lacZ M15 lacX74 recA1 ara 139 (ara-leu)7697 galU galK rpsL (StrR) endA1 nupG*	New England BioLabs

Table S7. Plasmids used in this study.

Plasmid	Description	Source
pBZ133	To construct *porA* $^{D142H\ E319Q\ E320Q}$ point mutant in *C. jejuni* 81-176, Cm^{R}.	This study
pBZ136	To construct *acpP* ^{G33R} point mutant in *C. jejuni* 11168, Kan^{R}.	This study
pBZ137	To construct *acpP* ^{A34P} point mutant in *C. jejuni* 81-176, Kan^{R}.	This study
pBZ141	To construct *porA* ^{D142H} point mutant in *C. jejuni* 81-176, Cm^{R}.	This study
pBZ142	To construct *porA* ^{E319Q} point mutant in C. *jejuni* 81-176, Cm^{R}.	This study
pBZ143	To construct *porA* ^{E320Q} point mutant in *C. jejuni* 81-176, Cm^{R}.	This study
pBZ156	To construct *porA* $^{E319Q\ E320Q}$ point mutant in *C. jejuni* 81-176, Cm^{R}.	This study

Table S8. Primers used in this study.

Primers	Oligosaccharides	Source
CS-1038 (NdeI-KO *porA* down arm-F)	GCACATATGGAAGCTTTCAAGTCTAACTTCAAG	This study
CS-1048 (MluI-KO *porA* down arm-R)	GCAACGCGTGGTACTAGAGGGGATATGTATGTTAAG	This study
CS-1094 (ApaI-*porA*-F)	GCAGGGCCCATGAAACTAGTTAAACTTAG	This study
CS-1034 (SphI-*porA*-R)	GCAGCATGCTTAGAATTTGTAAAGAGCTTGAAG	This study
CS-1012 (*porA* ^{D142H} GAT to CAT-F)	GGACTGACAATGGAGTTCATGGTTTAGTAGGAACAGGTATC	This study
CS-1013 (*porA* ^{D142H} GAT to CAT-R)	CCTGTTCCTACTAAACCATGAACTCCATTGTCAGTCCAGATAG	This study
CS-1014 (*porA* ^{E319Q} GAG to CAG-F)	GGTTCTTTACTTGCAGGTCAGGAAATTTTCTATACTACTGGTTC	This study
CS-1015 (*porA* ^{E319Q} GAG to CAG-R)	GTAGTATAGAAAATTTCCTGACCTGCAAGTAAAGAACCAAG	This study
CS-1016 (*porA* ^{E320Q} GAA to CAA-F)	CTTTACTTGCAGGTGAGCAAATTTTCTATACTACTGGTTCAAG	This study
CS-1017 (*porA* ^{E320Q} GAA to CAA-R)	CCAGTAGTATAGAAAATTTGCTCACCTGCAAGTAAAGAACCAAG	This study
CS-1096 (*porA* $^{E319Q\ E320Q}$ GAGGAA to CAGCAA-F)	GGTTCTTTACTTGCAGGTCAGCAAATTTTCTATACTACTGGTTC	This study
CS-1097 (*porA* $^{E319Q\ E320Q}$ GAGGAA to CAGCAA R)	CCAGTAGTATAGAAAATTTGCTGACCTGCAAGTAAAGAACCAAG	This study
CS-1024 (AatII-mutate *acpP* up arm-F)	GCAGACGTCAGCTGATTGTGTTGTAACACCTTG	This study
CS-1095 (NcoI-*acpP*-R)	GCACCATGGTTATTTTTTTAGATTGTCAATATAATTTAC	This study
CS-1026 (SpeI- mutate *acpP* down arm-F)	GCAACTAGTTTTTTTTTCTTGCAAGGAGCTTATTC	This study
CS-1027 (NdeI- mutate *acpP* down arm-R)	GCACATATGGAACTTATAAGCGGGATATTATTGC	This study

References

1. Garber JM. 2019. Characterization of carbohydrate metabolism in *Campylobacter jejuni*. Ph.D. thesis. University of Georgia, Athens, GA.
2. Yan A, Butcher J, Schramm L, Mack DR, Stintzi A. 2023. Multiomic spatial analysis reveals a distinct mucosa-associated virome. Gut Microbes 15:2177488.
3. Palyada K, Threadgill D, Stintzi A. 2004. Iron acquisition and regulation in *Campylobacter jejuni*. J Bacteriol 186:4714-29.
4. Kopylova E, Noe L, Touzet H. 2012. SortMeRNA: fast and accurate filtering of ribosomal RNAs in metatranscriptomic data. Bioinformatics 28:3211-7.
5. Langmead B, Salzberg SL. 2012. Fast gapped-read alignment with Bowtie 2. Nat Methods 9:357-9.
6. Anders S, Pyl PT, Huber W. 2015. HTSeq--a Python framework to work with high-throughput sequencing data. Bioinformatics 31:166-9.
7. Love MI, Huber W, Anders S. 2014. Moderated estimation of fold change and dispersion for RNA-seq data with DESeq2. Genome Biol 15:550.
8. Sacher JC, Javed MA, Crippen CS, Butcher J, Flint A, Stintzi A, Szymanski CM. 2021. Reduced Infection Efficiency of Phage NCTC 12673 on Non-Motile *Campylobacter jejuni* Strains Is Related to Oxidative Stress. Viruses 13.
9. Sacher JC, Shajahan A, Butcher J, Patry RT, Flint A, Hendrixson DR, Stintzi A, Azadi P, Szymanski CM. 2020. Binding of Phage-Encoded FlaGrab to Motile *Campylobacter jejuni* Flagella Inhibits Growth, Downregulates Energy Metabolism, and Requires Specific Flagellar Glycans. Front Microbiol 11:397.

10. Totten SM, Wu LD, Parker EA, Davis JC, Hua S, Stroble C, Ruhaak LR, Smilowitz JT, German JB, Lebrilla CB. 2014. Rapid-throughput glycomics applied to human milk oligosaccharide profiling for large human studies. Anal Bioanal Chem 406:7925-35.

11. Anumula KR, Taylor PB. 1992. A comprehensive procedure for preparation of partially methylated alditol acetates from glycoprotein carbohydrates. Anal Biochem 203:101-8.

12. Parkhill J, Wren BW, Mungall K, Ketley JM, Churcher C, Basham D, Chillingworth T, Davies RM, Feltwell T, Holroyd S, Jagels K, Karlyshev AV, Moule S, Pallen MJ, Penn CW, Quail MA, Rajandream MA, Rutherford KM, van Vliet AH, Whitehead S, Barrell BG. 2000. The genome sequence of the food-borne pathogen Campylobacter jejuni reveals hypervariable sequences. Nature 403:665-8.

13. Korlath JA, Osterholm MT, Judy LA, Forfang JC, Robinson RA. 1985. A point-source outbreak of campylobacteriosis associated with consumption of raw milk. J Infect Dis 152:592-6.
