# Supplementary material for: Campylobacter jejuni resistance to human milk involves the acyl carrier protein AcpP
Source: mBio. 2025 Feb 25;16(4):e03997-24. doi: 10.1128/mbio.03997-24 (PMC11980577; doi:10.1128/mbio.03997-24)
Supplement: Captions — Details for Tables S1-S4. [file mbio.03997-24-s0008.pdf]

Supplemental material –Tables S1-S4

Campylobacter jejuni resistance to human milk involves the acyl carrier protein AcpP

Bibi Zhou[a,b], Jolene M. Garber[a,b*], James Butcher[c], Artur Muszynski[b], Rebekah L. Casey[d], Steven Huynh[e], Stephanie Archer-Hartmann[b], Sara Porfirio[b], Ashley M. Rogers[a,b], Parastoo Azadi[b], Craig T. Parker[e], Kenneth K. S. Ng[f], Kelly M. Hines[d], Alain Stintzi[c] and Christine M. Szymanski[a,b#]

[a]Department of Microbiology, University of Georgia, Athens, GA, USA.

[b]Complex Carbohydrate Research Center, University of Georgia, Athens, GA, USA.

[c]School of Pharmaceutical Sciences, Ottawa Institute of Systems Biology and Department of Biochemistry, Microbiology and Immunology, Faculty of Medicine, University of Ottawa, Ottawa, Ontario, Canada.

[d]Department of Chemistry, University of Georgia, Athens, GA, USA.

[e]Agricultural Research Service, U.S. Department of Agriculture, Produce Safety and Microbiology Research Unit, Albany, CA, USA.

[f]Department of Chemistry and Biochemistry, University of Windsor, Windsor, ON, Canada

Table S1, Excel file, Transcript profiles of *C. jejuni* strains 11168 and 81-176 in human milk
Table S2, Excel file, MS profiles of human milk oligosaccharides used in this study
Table S3, Excel file, LC-MS of 81-176 lipids (PG, PE, LPE and FA)
Table S4, Excel file, LC-MS of 11168 lipids (PG, PE, LPE and FA)
