# Supplementary material for: Campylobacter jejuni resistance to human milk involves the acyl carrier protein AcpP
Source: mBio. 2025 Feb 25;16(4):e03997-24. doi: 10.1128/mbio.03997-24 (PMC11980577; doi:10.1128/mbio.03997-24)
Supplement: Table S5 — LL-37. [file mbio.03997-24-s0010.pdf]

Supplemental material – Methods and Table S5

Campylobacter jejuni resistance to human milk involves the acyl carrier protein AcpP

Bibi Zhou[a,b], Jolene M. Garber[a,b*], James Butcher[c], Artur Muszynski[b], Rebekah L. Casey[d], Steven Huynh[e], Stephanie Archer-Hartmann[b], Sara Porfirio[b], Ashley M. Rogers[a,b], Parastoo Azadi[b], Craig T. Parker[e], Kenneth K. S. Ng[f], Kelly M. Hines[d], Alain Stintzi[c] and Christine M. Szymanski[a,b#]

[a]Department of Microbiology, University of Georgia, Athens, GA, USA.

[b]Complex Carbohydrate Research Center, University of Georgia, Athens, GA, USA.

[c]School of Pharmaceutical Sciences, Ottawa Institute of Systems Biology and Department of Biochemistry, Microbiology and Immunology, Faculty of Medicine, University of Ottawa, Ottawa, Ontario, Canada.

[d]Department of Chemistry, University of Georgia, Athens, GA, USA.

[e]Agricultural Research Service, U.S. Department of Agriculture, Produce Safety and Microbiology Research Unit, Albany, CA, USA.

[f]Department of Chemistry and Biochemistry, University of Windsor, Windsor, ON, Canada

Measurement of LL-37 minimum inhibitory concentration.

C. jejuni cells were harvested from MH agar plates using MH broth, the cell density was adjusted to OD_{600}=0.05, and 100µL of the cell suspension was distributed across a 96-well plate. LL-37 was added to the wells starting from 10µg/mL and 2-fold serially diluted across the plate. The plate was incubated at 37°C under microaerobic conditions overnight and growth was assessed by OD_{600}. The MIC was determined by observing the first well where growth was no longer detected.

Table S5. Minimum inhibitory concentration (MIC) of LL-37 on *C. jejuni* 11168, *C. jejuni* 11168E, *C. jejuni* 81-176, and *C. jejuni* 81-176E.

Strains	LL-37 MIC (µg/mL)
C. jejuni 11168	10
C. jejuni 11168E	10
C. jejuni 81-176	10
C. jejuni 81-176E	10
